# A Novel HIV-1 RNA Testing Intervention to Detect Acute and Prevalent HIV Infection in Young Adults and Reduce HIV Transmission in Kenya: Protocol for a Randomized Controlled Trial

**DOI:** 10.2196/16198

**Published:** 2020-08-07

**Authors:** Susan M Graham, Clara Agutu, Elise van der Elst, Amin S Hassan, Evanson Gichuru, Peter M Mugo, Carey Farquhar, Joseph B Babigumira, Steven M Goodreau, Deven T Hamilton, Thumbi Ndung'u, Martin Sirengo, Wairimu Chege, Eduard J Sanders

**Affiliations:** 1 Department of Global Health University of Washington Seattle, WA United States; 2 Department of Medicine University of Washington Seattle, WA United States; 3 Department of Epidemiology University of Washington Seattle, WA United States; 4 Kenya Medical Research Institute - Wellcome Trust Research Programme Kilifi Kenya; 5 Department of Pharmacy University of Washington Seattle, WA United States; 6 Department of Anthropology University of Washington Seattle, WA United States; 7 Center for Studies in Demography and Ecology University of Washington Seattle, WA United States; 8 Africa Health Research Institute Durban South Africa; 9 HIV Pathogenesis Programme Doris Duke Medical Research Institute University of KwaZulu-Natal Durban South Africa; 10 Max Planck Institute for Infection Biology Berlin Germany; 11 Division of Infection and Immunity University College London London United Kingdom; 12 Department of Health Infrastructure Management Ministry of Health Nairobi Kenya; 13 Division of AIDS National Institute of Allergy and Infectious Diseases National Institutes of Health Rockville, MD United States; 14 University of Oxford Headington United Kingdom

**Keywords:** diagnostic tests, HIV infection, viral burden, contact tracing, highly active antiretroviral therapy, pre-exposure prophylaxis

## Abstract

**Background:**

Detection and management of acute HIV infection (AHI) is a clinical and public health priority, and HIV infections diagnosed among young adults aged 18 to 39 years are usually recent. Young adults with recent HIV acquisition frequently seek care for symptoms and could potentially be diagnosed through the health care system. Early recognition of HIV infection provides considerable individual and public health benefits, including linkage to treatment as prevention, access to risk reduction counseling and treatment, and notification of partners in need of HIV testing.

**Objective:**

The Tambua Mapema Plus study aims to (1) test 1500 young adults (aged 18-39 years) identified by an AHI screening algorithm for acute and prevalent (ie, seropositive) HIV, linking all newly diagnosed HIV-infected patients to care and offering immediate treatment; (2) offer assisted HIV partner notification services to all patients with HIV, testing partners for acute and prevalent HIV infection and identifying local sexual networks; and (3) model the potential impact of these two interventions on the Kenyan HIV epidemic, estimating incremental costs per HIV infection averted, death averted, and disability-adjusted life year averted using data on study outcomes.

**Methods:**

A modified stepped-wedge design is evaluating the yield of this HIV testing intervention at 4 public and 2 private health facilities in coastal Kenya before and after intervention delivery. The intervention uses point-of-care HIV-1 RNA testing combined with standard rapid antibody tests to diagnose AHI and prevalent HIV among young adults presenting for care, employs HIV partner notification services to identify linked acute and prevalent infections, and follows all newly diagnosed patients and their partners for 12 months to ascertain clinical outcomes, including linkage to care, antiretroviral therapy (ART) initiation and virologic suppression in HIV-infected patients, and pre-exposure prophylaxis uptake in uninfected individuals in discordant partnerships.

**Results:**

Enrollment started in December 2017. As of April 2020, 1374 participants have been enrolled in the observation period and 1500 participants have been enrolled in the intervention period, with 13 new diagnoses (0.95%) in the observation period and 37 new diagnoses (2.47%), including 2 AHI diagnoses, in the intervention period. Analysis is ongoing and will include adjusted comparisons of the odds of the following outcomes in the observation and intervention periods: being tested for HIV infection, newly diagnosed with prevalent or acute HIV infection, linked to care, and starting ART by week 6 following HIV diagnosis. Participants newly diagnosed with acute or prevalent HIV infection in the intervention period are being followed for outcomes, including viral suppression by month 6 and month 12 following ART initiation and partner testing outcomes.

**Conclusions:**

The Tambua Mapema Plus study will provide foundational data on the potential of this novel combination HIV prevention intervention to reduce ongoing HIV transmission in Kenya and other high-prevalence African settings.

**Trial Registration:**

ClinicalTrials.gov NCT03508908; https://clinicaltrials.gov/ct2/show/NCT03508908

**International Registered Report Identifier (IRRID):**

DERR1-10.2196/16198

## Introduction

While some patients with acute HIV infection (AHI) remain asymptomatic, most experience an acute illness approximately 2 weeks following infection, and the majority of these patients seek urgent care [1-5]. Common symptoms of AHI include fever, joint and muscle pains, headache, fatigue, and rash, while a minority of patients have a mononucleosis-like illness with fever, sore throat, and oral ulcers [6]. In coastal Kenya, 81% of female sex workers participating in a prospective cohort experienced symptoms with seroconversion and 44% were sick enough to prevent them from working [7]. In a nearby Kenyan cohort of adults at high risk for HIV-1 acquisition, 69% of those who seroconverted had sought care at the research clinic or elsewhere due to AHI symptoms [3]. Unfortunately, most individuals who seek care in the setting of HIV-1 acquisition have not yet developed antibodies and are missed by standard HIV rapid antibody tests [8,9].

The 2012 Kenyan AIDS Indicator Survey showed a steady increase in HIV prevalence with increasing age among adults, with HIV prevalence peaking in women aged 35 to 39 years and men aged 45 to 49 years [10]. Given these data, our research team posited that identifying young adults aged 18 to 39 with AHI would be a key opportunity to interrupt ongoing transmission. Due to very high viral loads and characteristics of the infecting viral strain, AHI is a period of heightened risk for secondary HIV-1 transmission [11-14]. The proportion of transmission attributed to AHI varies with epidemic stage and other local factors but has been estimated to range from 25% to 50% in studies using viral sequences. Unfortunately, no current HIV-1 prevention guidelines in sub-Saharan Africa recommend evaluation for AHI among young adult patients seeking care who test negative on standard rapid antibody tests [15].

While guidance exists for persons with discordant rapid antibody test results (ie, repeat testing is recommended after 2 weeks) [16], the value of demographic factors, signs, and symptoms to target AHI testing in patients with negative serologic test results has been unclear. In a study using cohort data from 4 sites in Kenya, Malawi, and South Africa, 122 AHI visits (ie, visits in which HIV-1 RNA or p24 antigen were detected in a seronegative patient who subsequently converted) were compared to 45,961 uninfected patient visits. In generalized estimating equation (GEE) modeling including signs and symptoms, age group, sex, and site, younger age (18-29 years) and reported fever, fatigue, body pains, diarrhea, sore throat, and genital ulcer disease (GUD) were independent predictors of AHI [17]. An AHI risk score was created that assigned a model-based score to each predictor, then calculated an overall risk score for each participant; GUD received a score of 3, while all other predictors received a score of 1 [17]. The performance (ie, area under the curve) for this AHI risk score overall was 0.78, with site-specific area under the curve estimates ranging from 0.61 to 0.89 [17]. A risk score of 2 or higher would indicate HIV-1 RNA testing for 15%, 26%, 50%, and 5% of risk populations in Mombasa and Kilifi, Kenya; Lilongwe, Malawi; and Durban, South Africa, respectively [17]. Sensitivity was highest for the risk score in Kilifi and Lilongwe (90.0% in Kilifi and 92.9% in Lilongwe), where the AHI risk score improved AHI detection over the published algorithms from these two sites [18,19].

In our earlier pilot study of AHI detection in Kenya, entitled Tambua Mapema (Kiswahili for “discover early”), all patients aged 18 to 29 years who met the AHI risk score criteria described above and had negative or discordant rapid antibody test results were tested using a p24 antigen assay, then underwent repeat rapid antibody testing for HIV infection 2 weeks after first presentation [20]. This pilot study (ClinicalTrials.gov NCT01876199) was conducted from April 2013 to July 2013 at a network of 5 health facilities and 5 pharmacies selected from the 26 health facilities and 26 pharmacies located in the study area, Mtwapa/Shanzu town (total population approximately 100,000) [20]. AHI was diagnosed in 5 of 506 patients with negative or discordant rapid antibody test results who met risk criteria and were completely evaluated, for an AHI prevalence of 1.0%. Of the 5 AHI cases, 4 were diagnosed among the 241 patients with a documented fever (prevalence 1.7%), versus 1 among the 265 nonfebrile patients (prevalence 0.4%; *P*=.15) [20].

Now that point-of-care (POC) RNA diagnostics designed for AHI detection are becoming available, real-time AHI diagnosis before the patient leaves the clinic is within reach [21,22]. Guidance on the potential impact of targeted HIV-1 RNA or p24 antigen testing programs on the HIV epidemic in sub-Saharan Africa is therefore needed. Use of a simple algorithm based on 7 features (aged 18-29 years, fever, fatigue, diarrhea, body pains, sore throat, and GUD) to target young adults for AHI testing could substantially reduce the number of symptomatic HIV-1–seronegative patients requiring HIV-1 RNA or p24 antigen testing, while still capturing most infections among persons who present for clinical care [17]. When paired with standard rapid HIV testing to diagnose prevalent HIV infection, such a testing intervention could greatly reduce transmission among young, sexually active adults.

The Tambua Mapema Plus study aims to use an AHI risk score to identify young (aged 18-39 years), previously HIV-negative or status-unknown adults presenting to health facilities for care to undergo an HIV testing intervention using POC HIV-1 RNA followed by rapid antibody testing to differentiate acute from prevalent infection. We hypothesize that targeted evaluation for AHI among young adults who have symptoms compatible with our AHI risk score will increase rates of case finding and linkage to care relative to standard provider-initiated testing and counseling (PITC), which has not been successful at targeting this group [15]. We also hypothesize that the use of World Health Organization–recommended assisted HIV partner notification services [23] will identify additional cases of previously undiagnosed HIV infection, including small outbreaks in local sexual networks, and that enhanced HIV partner notification services using HIV-1 RNA testing will identify more infected partners than standard HIV partner notification services using rapid antibody tests. Finally, we hypothesize that the identification of previously undiagnosed acute and prevalent HIV infections will lead to a significant reduction in new HIV infections in Kenya and will be cost-effective under a range of assumptions.

## Methods

### Study Overview

Tambua Mapema Plus is a proof-of-concept study to determine outcomes of our health facility–based HIV-1 RNA testing intervention to identify acute (ie, RNA positive, seronegative or discordant rapid antibody test results) and prevalent (ie, RNA positive, seropositive) HIV infection compared with standard care. A related objective has been to conduct focus group discussions with up to 60 individuals who work in the 6 health facilities where the trial has taken place (up to 10 participants per facility) to obtain their views on HIV-1 RNA testing and the research carried out at the facility, including challenges to intervention scale-up.

Secondary objectives for all individuals newly diagnosed with HIV infection during the study include (1) linkage to care and immediate antiretroviral therapy (ART), (2) partner testing, (3) barriers and facilitators, and (4) impact and cost-effectiveness.

First, we will provide linkage to care and immediate ART to determine the feasibility, acceptability, and uptake of offering immediate linkage and ART to all newly diagnosed patients with HIV in the intervention period, comparing this approach to standard care.

Second, we will conduct partner testing to determine the feasibility, acceptability, and uptake of HIV partner notification services for partner identification and testing, comparing the enhanced HIV partner notification services and HIV-1 RNA testing in the intervention period to the passive referral followed by delayed HIV partner notification services with standard HIV testing in the observation period.

Third, to identify barriers and facilitators, we will conduct qualitative in-depth interviews with up to 60 newly diagnosed patients with prevalent HIV or AHI and seronegative partners in discordant relationships to gain insights into their HIV testing experience and subsequent intervention uptake, including barriers and facilitators to ART or pre-exposure prophylaxis (PrEP) uptake and adherence in these groups.

Fourth, we will model the potential impact and cost-effectiveness of the HIV-1 RNA testing, linkage, immediate treatment, and partner notification interventions on the Kenyan HIV epidemic in terms of incremental costs per HIV infection averted, death averted, and disability-adjusted life years (DALYs) averted, using data on standard care outcomes from the observation period and data on intervention outcomes from the intervention period.

Full details on the procedures for these secondary objectives are included in Multimedia Appendix 1.

This protocol was prepared in accordance with Standard Protocol Items: Recommendations for Interventional Trials guidelines and is registered with the National Institutes of Health Division of AIDS Protocol Registration Office (DAIDS-ES Document Number: 38181) and with ClinicalTrials.gov (NCT03508908). All consents and additional details not found in this summary protocol can be found in the full published protocol.

### Study Design

A modified stepped-wedge design has been used to evaluate the yield of the HIV-1 RNA testing intervention at 4 public and 2 private health facilities in Kenya before (1375 patients) and after (1500 patients) intervention delivery. We chose a modified stepped-wedge trial design for the following reasons: (1) we predicted that the intervention would do more good than harm and (2) for logistical reasons, it was not practical to deliver the intervention simultaneously to all participants. Rolling out the intervention in a staggered fashion ensured that there was adequate time for individual site preparation and staff training as well as oversight of study activities. This study has therefore been conducted in 2 phases at each site.

#### Observation Period

The first phase was an observation period, in which all testing and treatment was conducted per Kenyan Ministry of Health guidelines and primary care clinician judgment. Young adults aged 18 to 39 years seeking care at primary health care clinics who had never been diagnosed with HIV and had a risk score of 2 or higher were offered participation. HIV testing was only done if ordered by the primary care clinician and was carried out according to standard care in Kenya, which currently misses AHI cases. Research procedures in the observation period consisted of a computer-assisted self-interview (CASI)/computer-assisted personal interview (CAPI) at baseline for all participants. Participants found to be HIV negative and those not tested ended their participation at the baseline visit. Those diagnosed HIV positive had a follow-up home visit at 6 weeks. The 6-week visit included a second CASI/CAPI and an assessment of linkage to care and treatment and of partner notification. Those who had not yet notified partners at this time point were offered standard HIV partner notification services [23]. Partners of observation phase HIV-positive participants (ie, index patients with prevalent HIV infection) were offered referrals for risk reduction counseling and for HIV care, if infected.

#### Intervention Period

In the intervention period, two major interventions were evaluated: testing for acute and prevalent HIV infection (primary objective) and enhanced HIV partner notification services using our HIV-1 RNA testing intervention (secondary objective). For the HIV-1 RNA testing intervention, a blood sample was obtained and tested for AHI using the Xpert HIV Qual assay (Cepheid Inc), with testing conducted on-site at the health facility where the participant was recruited and enrolled. This assay has been found to be easy to use and feasible in a community-based facility with limited or no laboratory infrastructure [24]. For participants in whom HIV-1 RNA was detected, a laboratory technician conducted rapid antibody testing (currently Determine; Abbott Laboratories and First Response; Premier Medical Corp) in accordance with Kenyan HIV testing guidelines. Test results were provided to participants in real time, with posttest counseling by research staff and detailed documentation of results.

As in the observation period, young adults aged 18 to 39 years seeking care at primary care clinics who had no history of HIV and who had a risk score of 2 or higher were offered enrollment into the intervention period. All intervention participants underwent the HIV testing intervention, which consisted of testing for HIV-1 RNA initially, followed (if positive) by 2 rapid antibody tests. Those who tested HIV negative ended participation at the baseline visit after the CASI/CAPI, as above. Those who tested HIV positive (either AHI or prevalent case) were offered enhanced HIV partner notification intervention and linkage to an ART cohort at Kenya Medical Research Institute (KEMRI) with 12 months of follow-up. Those who declined follow-up at KEMRI ended their participation at a 6-week home visit (as described above for the observation period). Those who tested HIV positive were also offered participation in qualitative interviews, which they could accept or decline without influencing other components of the study.

Partners of intervention phase participants with HIV (ie, index patients with newly diagnosed acute and prevalent HIV infection) were offered the HIV-1 RNA testing intervention (as described for participants in the intervention phase above) and enrollment into the KEMRI ART cohort or a PrEP cohort with 12 months of follow-up, depending on their test results. In the intervention period, partners newly diagnosed with HIV through the enhanced HIV partner notification intervention were also offered the enhanced HIV partner notification intervention to identify and test their partners. Identified partners of these individuals were offered the same options as partners of index patients diagnosed in the stepped-wedge trial.

#### Study Sites

**Figure 1:**
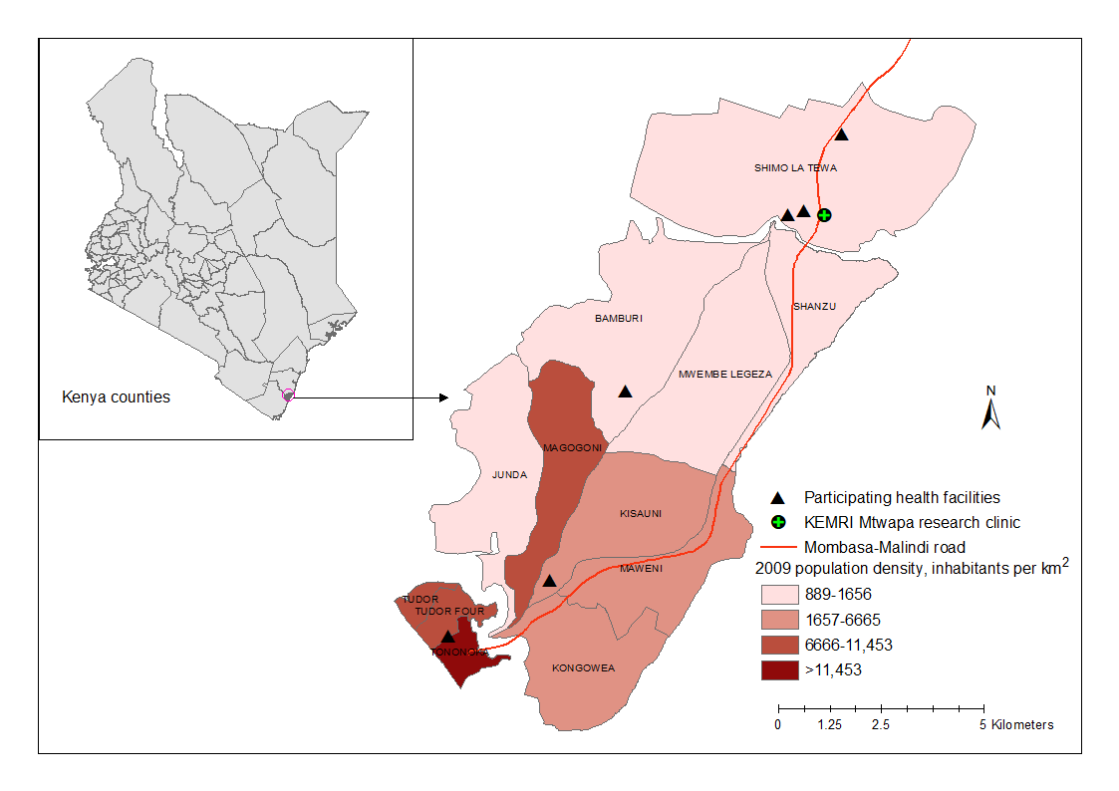
Map of Tambua Mapema Plus study sites.

Our HIV-1 RNA testing intervention network included both public and private health facilities in a large periurban area (population >100,000) in coastal Kenya (Figure 1). This area, known for its busy nightlife, sex work, and tourist industry, has been the site of a KEMRI HIV/sexually transmitted infection research clinic since 2005. We selected 6 health facilities (4 public, 2 private) for inclusion in this study due to their size, central location (within 20 kilometers of our KEMRI research clinic in Mtwapa), patient volume (>500 patients aged 18-39 years seen over 3 months), availability of HIV rapid antibody testing on-site, availability of a private room for consenting participants, and willingness to collaborate with the research team.

#### Randomization

**Figure 2:**
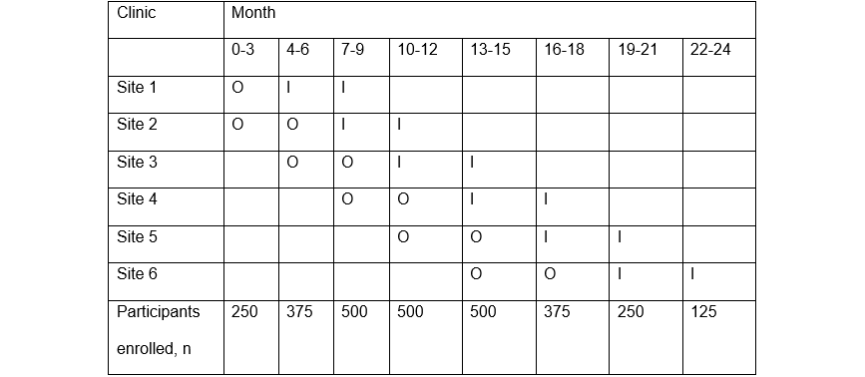
Stepped-wedge design at 6 sites. I: intervention; O: observation.

Before study initiation, the 6 participating clinics were randomized into observation and intervention periods, as presented in Figure 2. Opening of the observational phase at each site was staggered in 3-month intervals so that no more than two sites would be in each phase at any given time. Each phase lasted 6 months per site, with the exception of the first site, which had only 3 months of observation to allow the study to proceed more efficiently.

Randomization was performed using a random number generator to assign each clinic to one of the 6 positions, with stratification by facility type (public vs private). Due to practical limitations, allocations were unblinded. We recruited 125 participants per clinic per 3-month time block, for 2875 total participants (1375 in the observation period and 1500 in the intervention period). By the end of the trial, each facility has undergone an observation period of 3 to 6 months and received the intervention for 3 to 6 months. Thus, each health facility or cluster acts as its own control, with comparison of the patients enrolled during the observation period with those enrolled during the intervention period. Using this design, we can model the effect of time on the intervention, adjusting for temporal variations in HIV incidence [25,26].

### Inclusion and Exclusion Criteria for the Stepped-Wedge Trial

Eligibility criteria for participation in the stepped-wedge trial included (1) aged 18 to 39 years; (2) not previously diagnosed with HIV infection; and (3) a score of 2 or higher on our risk score algorithm [17] used to identify persons at higher risk for AHI, with scoring as follows: aged 18 to 29 years (1 point), fever (1 point), fatigue (1 point), body pains (1 point), diarrhea (1 point), sore throat (1 point), and GUD (3 points).

Eligibility criteria for partners of newly diagnosed patients with prevalent or acute HIV included (1) aged 18 years or older and (2) not previously diagnosed with HIV infection. Patients not meeting inclusion criteria or those who were not willing or able to participate (eg, due to illness or time constraints, or at the discretion of the study clinician) were excluded. Individuals at high risk for intimate partner violence (IPV) were excluded from the enhanced HIV partner notification intervention but eligible for all other components of the study. While sex work and other high-risk sexual behaviors were not part of the eligibility criteria for the study, data on sexual risk behavior were collected from all eligible patients.

### Inclusion Criteria for Staff Focus Group Discussions

Focus groups were held for up to 60 staff members at the 6 participating health facilities (up to 10 participants per facility). These individuals could work in any role but were required to have the following characteristics in order to participate: aged 18 years or older, planning to remain with the facility for the duration of trial implementation at that site, and willing to provide views on the detection of AHI and the research carried out at the facility, including challenges to intervention scale-up. Most participants were medical or clinical officers, nurses, laboratory technicians, or counselors.

### Recruitment for the Stepped-Wedge Trial

Eligible participants were recruited from among young (aged 18-39 years) adults who presented to any of the 6 health facilities in our HIV testing network. At each facility, we aimed to enroll 2 to 4 participants per day, for a minimum of 10 and maximum of 20 participants per week. Staffing schedules were developed in collaboration with each health facility, with a goal to ensure that recruitment targets were attained and that a range of time periods (eg, daytime, evening, weekend) were covered. While research staff were present at the facility, patients in the target age range were approached consecutively for study screening.

After obtaining verbal permission from the patient, facility clinicians or research staff present in the clinical room screened patients to determine eligibility and asked if they were willing to discuss participation with the research team after their consultation. The screening form (Multimedia Appendix 2) included a permission script to be read to potential participants and a space for research staff to initial that verbal consent for screening was provided. If a patient refused screening, only sex and estimated age were recorded; no identifying information was obtained during screening. All screening outcomes (ie, screening refused, screened out, screened in but refused participation, screened in and consented) were documented using the screening form. Facility clinicians then provided symptom-directed treatment to patients as per standard care and current Kenyan guidelines.

Upon completion of the consultation and before any HIV testing was conducted, patients who met eligibility criteria were approached by the research team and invited to participate in the study. Individuals recruited during the intervention period were shown a 2-minute, institutional review board–approved explainer video that presented the study rationale and overview in English or in Kiswahili [27]. Patients were then consented by research staff, who explained the purpose and design of the study, taking care to inform patients that participation was voluntary and would not influence their access to diagnostic testing or care. 

### Stepped-Wedge Trial and Procedures for Newly Diagnosed Participants

**Figure 3:**
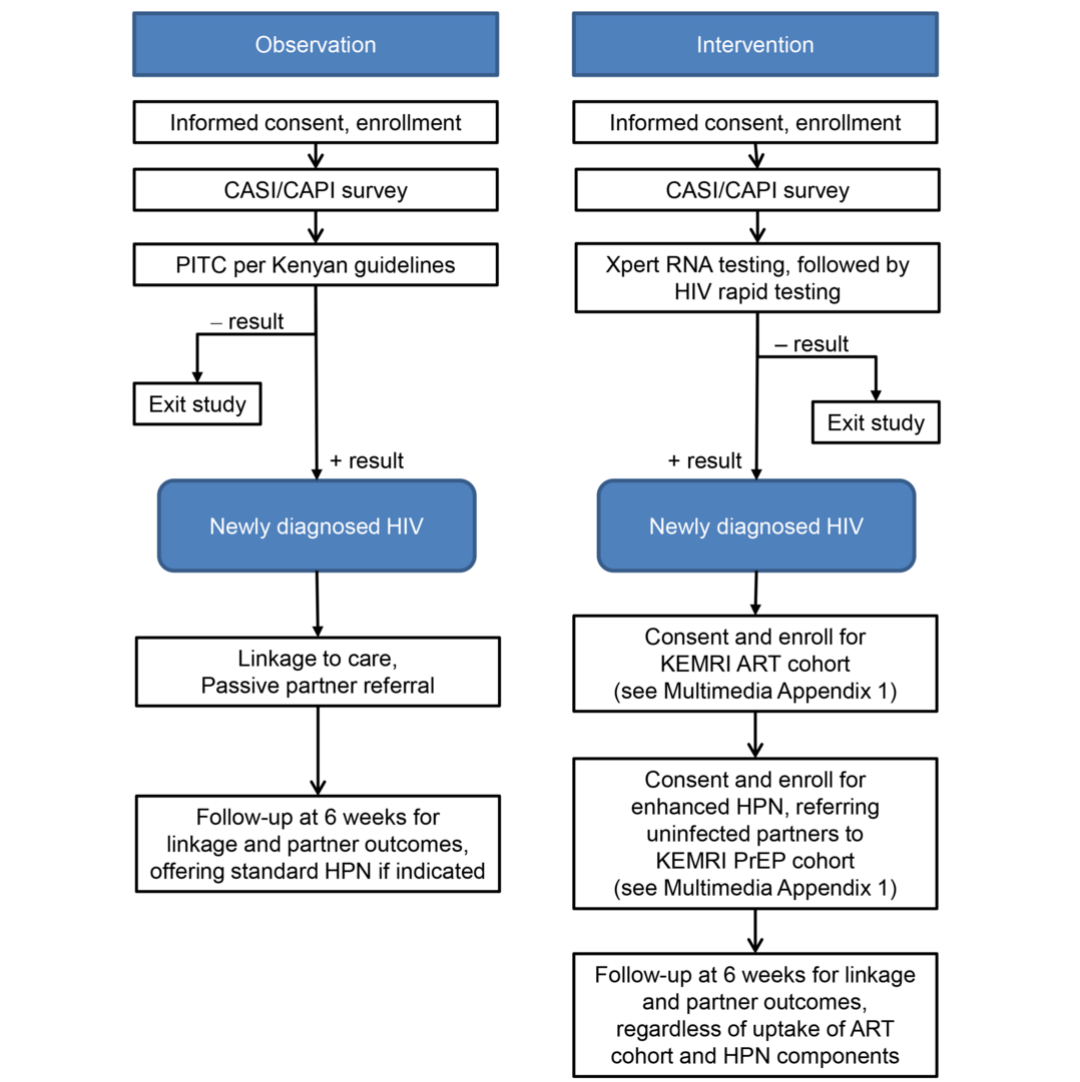
Flow diagram of observation and intervention periods. ART: antiretroviral therapy; CAPI: computer-assisted personal interview; CASI: computer-assisted self-interview; HPN: HIV partner notification services; KEMRI: Kenya Medical Research Institute; PITC: provider-initiated testing and counseling; PrEP: pre-exposure prophylaxis.

Figure 3 provides a flow diagram of procedures in the observation period and intervention period. A schedule of procedures for each period can be found in Multimedia Appendix 1, which also details procedures for all newly diagnosed participants in the intervention period, including 12-month follow-up in an ART cohort with periodic in-depth interviews, as well as enhanced HIV partner notification. Uninfected regular partners of newly diagnosed study participants were invited to 12-month follow-up in a PrEP cohort with periodic in-depth interviews.

#### Observation Period: Enrollment Visit

Consenting patients took a brief CASI or CAPI (≤20 minutes) on a handheld tablet computer. This survey, included in Multimedia Appendix 3, captures demographic information, onset of illness, and data on sexual behavior, including partner numbers (with detailed questions on the 3 most recent partners), relational timing (ie, concurrent vs sequential), transactional sex, and same-sex behavior. After the CASI/CAPI, research staff answered any questions about the CASI/CAPI and offered counseling as needed, then helped participants who had a request form for PITC to find the laboratory. PITC results were recorded, and facility staff referred patients with a positive result to care, as per their standard practice. At the conclusion of their study visit, participants were asked about the last time they tested for HIV, as well as costs they incurred for the health facility visit. All study participants who were newly diagnosed with HIV in the study were asked for contact details in order to arrange a 6-week follow-up visit. Research staff stressed the importance of linking to care, the availability of ART regardless of cluster of differentiation 4 cell count, and the need to inform partners that they should be tested.

#### Observation Period: 6-Week Follow-Up Visit

Individuals with a new diagnosis of HIV infection made in the observation period were visited in person or seen at the health facility from which they were recruited 6 weeks after diagnosis to repeat the CASI/CAPI and ascertain data on linkage to care, ART status, and partner notification outcomes. Linkage was verified by demonstration of a clinic registration card, and ART status was confirmed by demonstration of a regimen card or the patient’s pills. Partner notification outcomes were self-reported by the index patient. Participants who had not linked to care, started ART, or disclosed their HIV status were offered counseling and referrals at this time. At the conclusion of this data collection and counseling, these index patients were offered standard HIV partner notification services [23]. For those who accepted, standard rapid antibody testing was offered to all identified and successfully contacted partners.

#### Intervention Period: Enrollment Visit

After consent was obtained, research staff drew a 4-mL blood sample from participants for intervention HIV testing (ie, HIV-1 RNA followed by rapid tests, as described above). During wait time for this testing (up to 90 minutes), participants took the brief CASI/CAPI survey described above. After the CASI/CAPI, research staff answered any questions about the CASI/CAPI and offered counseling as needed, then helped patients with an order for lab tests other than an HIV test to find the facility laboratory. HIV test results were provided to participants in real time, with posttest counseling by KEMRI research staff; results were also shared with the facility clinician. At the conclusion of their study visit, participants were asked about the last time they tested for HIV, as well as costs they incurred for the health facility visit. All study participants who were diagnosed with acute or prevalent HIV infection by the KEMRI research team were asked for contact details in order to arrange a 6-week follow-up visit. Linkage to KEMRI ART cohort participation or care at a nonresearch facility of their choice was also offered at this time, and an IPV assessment previously used in Kenya [28] was conducted to determine eligibility for the enhanced HIV partner notification intervention.

#### Intervention Period: 6-Week Follow-Up Visit

Individuals with a new diagnosis of HIV infection made in the intervention period were visited in person or seen at the KEMRI research clinic 6 weeks after diagnosis to repeat the CASI/CAPI and ascertain data on linkage to care, ART status, and partner notification outcomes. Linkage was verified by demonstration of a clinic registration card, and ART status was confirmed by demonstration of a regimen card or the patient’s pills. Partner notification outcomes were self-reported by the index patient. Of note, additional data were available on linkage, ART status, and partner notification outcomes for patients participating in the enhanced HIV partner notification intervention or the ART cohort. However, the 6-week follow-up visit served to document outcomes at the same time point in all trial participants, regardless of their uptake of other components of the Tambua Mapema Plus intervention package.

### Views of Health Facility Staff

We held focus group discussions with up to 60 facility staff (10 in a single group at each participating facility) before and after the intervention was conducted. KEMRI research staff scheduled focus group discussions at facilities or at an off-site location, depending on facility and staff preferences. Before the intervention was delivered, facility staff members were asked about their general views on HIV testing, who is usually targeted for testing, and constraints to testing in their clinic. After these topics were addressed, participants were asked about the importance of early detection of HIV infection and the prevention of transmission through finding and testing partners of newly diagnosed individuals. After the intervention, we asked participants about the impact the Tambua Mapema Plus study had on the health facility in general and any challenges encountered during the study. We also asked their views about what factors might make it easier or more difficult to scale up a similar intervention in other health facilities in Kenya. Focus group discussions were led by a KEMRI research team member, with a second member present for note taking. When participants agreed to this, focus group discussions were tape-recorded. Multimedia Appendix 4 includes the topics that guides used for focus group discussions before and after the intervention.

### Ethical Considerations

Risks of study participation consist mainly of social harms involving a breach of confidentiality. Especially with respect to the enhanced HIV partner intervention, IPV, or physical, sexual, or psychological harm by a current or former sexual partner, there is a potential risk at the initial health facility visit or at any time during cohort follow-up. The study team collects data on social harms, which are reported using a study-specific incident report form. In the event that a participant reports a social harm, every effort is made to provide appropriate care and counseling to the participant, either by the study team or through a referral as indicated. Research staff have been trained on counseling and the provision of referrals to counseling and social service support. While maintaining participant confidentiality, KEMRI may also engage their community representatives in exploring the social context surrounding instances of social harm in order to mitigate harm and minimize recurrences. Special monitoring on a weekly basis is undertaken for participants at moderate risk for IPV, and a protocol safety review team monitors for IPV. Stopping rules for the study will be activated if more than 3 episodes of physical IPV are reported at a single study site or more than 5 episodes are reported in the study overall.

This protocol was approved by the KEMRI Scientific and Ethics Review Unit (No. 3280), the University of Washington Human Subjects Division (STUDY00001808) and the Oxford Tropical Research Ethics Committee (Protocol 46-16). All participants provided written informed consent using a consent form specific to the relevant study component. Participants were reimbursed for study participation according to local norms in Kenya (KSh 350-500, or US $3.26-$4.68), depending on visit type and duration). A community advisory board provided study oversight, with input from local KEMRI community representatives at regular meetings.

### Sample Size Considerations

The trial is powered for the stepped-wedge design. Based on our pilot work, we estimated that 50% to 60% of adults aged 18 to 39 years at the 6 participating facilities would be eligible for the study, and approximately 50% to 80% of these would accept study participation. Our preliminary data showed that approximately 2% of young adults in this age range were diagnosed with prevalent HIV-1 infection under standard care [15], while approximately 5% were newly diagnosed with acute (1%) or prevalent (4%) HIV-1 infection when testing for both HIV-1 antigen and antibodies was routinely delivered [20]. If these estimates are correct, with 1375 participants in the observation period and 1500 participants in the intervention period and a type I error probability of 0.05, assuming a coefficient of variation (*k*) of 0.25, we will have more than 90% power to reject the null hypothesis that the HIV diagnosis rates for experimental and control subjects are equal [26]. This power is more than adequate for multivariable analysis related to the testing of this null hypothesis. Because facilities do not have the laboratory capacity to diagnose HIV before seroconversion during the observation period, the base rate of AHI detection is 0, and power to detect AHI during the intervention period is greater than 90% even if AHI prevalence is well below 1%. Table 1 presents power for a range of coefficients of variation and acute and prevalent HIV prevalence in the intervention period, given our chosen sample size. Accounting for correlation within sites (ie, facilities participating in the randomized trial), precision for the estimated rate of AHI detection is within 0.54%, assuming a prevalence of 1% and *k* of 0.25; if AHI prevalence is 2%, precision will be within 0.81%.

**Table 1:** Power for evaluation of null hypothesis that HIV diagnosis rates are equal.

Diagnosed HIV prevalence, observation period	Diagnosed HIV prevalence, intervention period	*k*	Power
2.0%	5.0%	0.20	94.4%
2.0%	4.3%	0.20	82.2%
2.0%	5.0%	0.25	93.0%
2.0%	4.3%	0.25	80.0%
2.0%	5.0%	0.30	91.4%
2.0%	4.3%	0.30	77.3%
2.0%	5.0%	0.35	89.4%
2.0%	4.3%	0.35	74.6%

### Data Analysis Plan

In general, *P* values of *P*<.05 will be considered significant; however, in analyses in which multiple null hypotheses are tested, *P* values will be adjusted using the Holm-Bonferroni method. Planned work to model the impact and cost-effectiveness of this HIV-1 RNA testing intervention is detailed in Multimedia Appendix 1.

#### HIV Testing Uptake and Diagnoses

We will compare the age and sex of individuals who accept versus refuse screening, using 2-tailed Student *t* and chi-square tests. In addition, we will tally reasons for refusal to participate among those eligible and compare eligible individuals who refuse versus consent to study enrollment, using chi-square or Fisher exact tests for categorical variables and Student *t* tests or Mann-Whitney *U* tests for continuous variables. Proportions of individuals who accept screening, are determined eligible, and enroll will be presented with exact binomial confidence limits. We will compare the proportion of patients with the following outcomes in the observation and intervention periods: (1) tested for HIV infection, (2) newly diagnosed with prevalent HIV infection (ie, HIV seropositive), and (3) newly diagnosed with AHI. Because we expect 0 AHI cases in the observation period, we will use a combined outcome (ie, newly diagnosed acute or prevalent HIV-1) to model the effect of the intervention so that models will converge. We will conduct analyses on the individual-level data, using log-binomial GEE models to account for clustering by health facility. We will use a small-sample variance correction to handle the small number of clusters [29]. Models will include indicator variables for calendar time period to control for trends in HIV incidence in the study area.

We will compare baseline characteristics between individuals in the observation and intervention groups, using chi-square or Fisher exact tests for categorical variables and Student *t* tests or Mann-Whitney *U* tests for continuous variables. Where imbalances are identified, we will control for these potential confounders in secondary analyses using GEE models, as above. We will also conduct exploratory analyses to identify predictors of the combined outcome (ie, newly diagnosed acute or prevalent HIV-1) in the intervention group, including age, sex, marital status, symptom or symptoms reported, sex of partners, number of partners, condom use, transactional sex work, and other risk behaviors. We will test for interactions between the intervention and other variables, such as sex, and will present stratified analyses if meaningful interaction is observed. 

#### Views of Health Facility Staff

Audiorecordings of the focus group discussions will be transcribed verbatim; identifying information will be omitted from transcripts. Transcribed focus group discussions will be entered into NVivo (QSR International), and analysis will aim to identify and categorize knowledge, attitudes, and contextual factors associated with PITC in general and the HIV-1 RNA testing intervention evaluated specifically. Views related to HIV testing compared with other facility laboratory testing, such as testing for malaria, will be identified. Data analysis will be iterative and include open coding, axial coding, marginal remarks, comparisons, and memo writing. Themes will be analyzed and triangulated using a grounded theory framework.

## Results

Study enrollment started in December 2017. As of April 2020, 1374 participants were enrolled in the observation period (1 participant was excluded due to a protocol violation) and 1500 participants were enrolled in the intervention period. During the observation period, 3368 of the 3382 patients approached (99.59%) accepted screening, of whom 1495 (44.39%) were eligible. Of the 1495 eligible patients, 1374 enrolled (91.91%). During the intervention period, 4889 of 4895 patients approached (99.88%) accepted screening, of whom 1818 (37.19%) were eligible. Of the 1818 eligible patients, 1500 enrolled (82.51%). There were 13 new HIV diagnoses (13/1374, 0.95% of those enrolled; 13/382, 3.4% of those tested) in the observation period and 37 new HIV diagnoses (37/1500, 2.47% of those enrolled and tested) in the intervention period. Of the 37 diagnoses in the intervention period, 2 were AHI diagnoses (5%). Linkage to care and ART by week 6 was successful for 9 of the 13 (69%) newly diagnosed patients in the observation period and for 33 of the 37 (89%) newly diagnosed patients in the intervention period. No IPV episodes related to study participation were reported by participants in either study period.

Analysis is ongoing and will include adjusted comparisons of the odds of being tested for HIV infection and of being newly diagnosed with prevalent or acute HIV infection by the HIV-1 RNA testing intervention, as well as adjusted comparisons of the odds of being linked to care and starting ART by week 6 following HIV diagnosis in the observation and intervention periods. Reporting will follow the recently published Consolidated Standards of Reporting Trials guidelines for stepped-wedge trials [30]. Qualitative analysis of staff focus groups will identify barriers and facilitators to facility-based HIV testing, views on the detection of AHI, and views on the HIV-1 RNA testing intervention carried out at the 6 health facilities, including challenges to intervention scale-up.

Follow-up in the ART and PrEP cohorts is ongoing and will end in March 2021. Additional analysis related to these cohorts will include evaluation of outcomes, including viral suppression by month 6 and month 12 following ART initiation, retention on and adherence to PrEP by month 6 and month 12 following PrEP initiation, and partner testing outcomes (ie, number of partners reported, successfully contacted, tested, newly diagnosed, and engaged in care with ART or PrEP as indicated). Qualitative analysis of participant interviews will identify barriers and facilitators to HIV testing uptake, HIV partner notification uptake, ART uptake and adherence, and PrEP uptake and adherence.

As cohort follow-up continues, modeling and cost-effectiveness analyses are planned, as detailed in Multimedia Appendix 1. Modeling outputs will include HIV infections averted, life years gained, DALYs averted, costs per HIV infection averted, costs per death averted, and costs per DALY averted. Impact and cost-effectiveness will be evaluated by comparing these outputs for standard care (observation period) to these outputs for the HIV-1 RNA testing intervention (intervention period).

## Discussion

In this stepped-wedge trial of a novel HIV-1 testing intervention, acceptance of screening was high (>99% in both periods; 3368/3382, 99.59% in the observation period and 4889/4895, 99.88% in the intervention period), and enrollment rates among those eligible was only somewhat lower in the intervention period (1500/1818, 82.51%), when HIV testing was performed for all patients, compared with the observation period (1374/1495, 91.91%), when decisions about HIV testing were left to the facility clinician. The proportion of participants newly diagnosed with HIV in the intervention period (37/1500, 2.47%) was higher than the proportion diagnosed in the observation period (13/1374, 0.95%), although it was lower than the proportion diagnosed among participants selected by providers for testing in the observation period (13/382, 3.4% of those who received PITC). There were 2 AHI cases diagnosed among the 37 total diagnoses in the intervention period, making up 5% of all cases diagnosed by the testing intervention. Linkage to care was high (33/37, 89%) in the intervention period, when intensive linkage to care procedures were in place, compared with linkage to care in the observation period (9/13, 69%), when standard referral was used. Data analysis that accounts for clustering within facilities is ongoing, and more detailed results will be presented by the end of 2020.

In the 2012 AIDS Indicator Survey, around the time this study was proposed, Kenya had an adult HIV prevalence of 5.6% [10], and most (53%) seropositive individuals were unaware of their status, presenting a major challenge for epidemic control [31]. Expanded testing efforts are critical in order to attain the Joint United Nations Programme on HIV/AIDS (UNAIDS) 90-90-90 targets, which stated that by 2020, 90% of all people living with HIV should know their HIV status, 90% of those who test positive should be provided therapy, and of those, 90% should achieve virologic suppression [32]. We believed that such outreach should take advantage of the care-seeking behavior of adults who acquire HIV-1 in sub-Saharan Africa. For example, in our study of 72 participants who acquired HIV-1 while participating in a prospective cohort in coastal Kenya, 54 (75%) reported fever and 50 (69%) had sought care for symptomatic illness, including 23 (32%) who sought care in a nonresearch setting [3]. Of note, 29 of the 72 (40%) patients received presumptive malaria treatment, suggesting that AHI is frequently misdiagnosed as malaria in Kenyan health facilities [3].

In the Tambua Mapema Plus study, we detected fewer AHI and prevalent HIV cases than predicted based on our earlier work. This may be a result of falling HIV incidence in Kenya, as the 2018 Kenya Population-based HIV Impact Assessment reported a decline in HIV incidence among adults aged 15 to 64 years from 0.5% in 2012 to 0.14% in 2018 [33]. Kenya has also made great progress on HIV testing; in its 2019 report, UNAIDS estimated that 89% of Kenyans of all ages who are living with HIV are aware of their status [34]. Of note, individuals known to have HIV infection were excluded from this study.

Detection and management of AHI has been called a “clinical and public health emergency” [14] and a “common occurrence overlooked” [35]. POC HIV-1 RNA testing is becoming more available in sub-Saharan Africa and has multiple clinical uses, including viral load monitoring, early infant diagnosis, and AHI detection [36]. Because the cost of HIV-1 RNA testing is considerable, detection of AHI in resource-limited countries should be targeted using algorithms that identify at-risk individuals [37]. The Tambua Mapema Plus study will provide foundational data on the potential of this novel combination HIV prevention intervention to reduce ongoing HIV transmission in Kenya and other high-prevalence African settings through the detection of AHI and prevalent HIV infection among young adults aged 18 to 39 years presenting to health facilities with symptoms of acute infectious illness. If our novel intervention proves cost-effective and promising in terms of reducing HIV-1 transmission, we will use these data to design further research focusing on implementation of the intervention, including barriers and facilitators to its success. If the use of HIV-1 RNA testing should prove too costly, we can still analyze the impact of scaling up rapid tests, which were part of our novel testing intervention. PITC at health facilities contributes the majority of new HIV diagnoses in most contexts in sub-Saharan Africa [38], yet innovations in health facility–based HIV testing are urgently needed to increase testing rates. Recently, the use of oral self-testing has been shown to increase HIV testing in health facilities [39]. It is our hope that analysis of the Tambua Mapema Plus trial data and our planned modeling and cost-effectiveness analysis efforts will make an important contribution to the optimization of facility-based HIV testing.

## Appendix

Multimedia Appendix 1 Detailed Procedures for Secondary Outcomes.

Multimedia Appendix 2 Tambua Mapema Plus Eligibility Form.

Multimedia Appendix 3 Computer-assisted self-interview/Computer-assisted personal interview.

Multimedia Appendix 4 Focus group topic guides.

## Abbreviations

AAS: African Academy of Sciences
AHI: acute HIV infection
ART: antiretroviral therapy
CAPI: computer-assisted personal interview
CASI: computer-assisted self-interview
DALY: disability-adjusted life year
DELTAS: Developing Excellence in Leadership, Training and Science
GEE: generalized estimating equation
GUD: genital ulcer disease
IPV: intimate partner violence
KEMRI: Kenya Medical Research Institute
NIAID: National Institute of Allergy and Infectious Diseases
PITC: provider-initiated testing and counseling
POC: point of care
PrEP: pre-exposure prophylaxis
UNAIDS: Joint United Nations Programme on HIV/AIDS
