# Supplementary material for: A Novel HIV-1 RNA Testing Intervention to Detect Acute and Prevalent HIV Infection in Young Adults and Reduce HIV Transmission in Kenya: Protocol for a Randomized Controlled Trial
Source: JMIR Res Protoc. 2020 Aug 7;9(8):e16198. doi: 10.2196/16198 (PMC7442943; doi:10.2196/16198)
Supplement: Multimedia Appendix 1 [file resprot_v9i8e16198_app1.docx]

# Multimedia Appendix 1: Detailed Procedures for Secondary Outcomes

## Schedules of Procedures

Detailed schedules of procedures for the observation period (Schedule 1), intervention period (Schedule 2), partner services (Schedule 3), antiretroviral therapy (ART) cohort (Schedule 4), and pre-exposure prophylaxis (PrEP) cohort (Schedule 5), are included at the end of this appendix.

## Recruitment Procedures

### Antiretroviral therapy cohort

Trial participants newly diagnosed with acute or prevalent HIV infection in the intervention period (estimated at 75 total) and any partners of these individuals who are newly diagnosed through partner testing are offered follow-up at the KEMRI Research Clinic. At the research clinic, these individuals are offered immediate ART (provided by the National AIDS and STI Control Programme) to prevent secondary transmission and adverse health outcomes, with follow-up for 12 months. If these individuals prefer not to participate in the KEMRI ART cohort, they are referred to any of several clinics offering HIV care, including ART, in the study area. Persons testing positive for HIV during the observation period are not eligible for the ART cohort, but are provided counseling and offered linkage to care.

### Enhanced HIV partner notification services intervention

All individuals newly diagnosed with HIV in the intervention period (both trial participants and partners) are offered participation in the enhanced HIV partner notification intervention (i.e., partner notification with testing for acute and prevalent HIV infection). Newly diagnosed individuals can opt out of enhanced HIV partner notification and still be followed in the ART cohort, according to their preference. During the observation period, standard HIV partner notification (i.e., partner notification with standard HIV testing) is offered to newly diagnosed individuals who have not yet informed their partners at the 6-week visit (detailed below). Counselors make it clear that participation in partner notification services involves providing contact information about recent sexual partners. Individuals who are not willing to disclose partner information are excluded from partner notification services.

### Partner testing and PrEP cohort

Partners identified through HIV partner notification services are offered HIV testing according to the period in which the associated index patient was diagnosed (i.e., standard rapid HIV tests in the observation period, testing for both acute and prevalent HIV infection in the intervention period). During the intervention period, partners who are newly diagnosed with HIV are offered enrolment in the ART cohort at the KEMRI Research Clinic and are also offered participation in the enhanced HIV partner notification intervention. Also in the intervention period, partners who are uninfected and in an ongoing serodiscordant partnership are offered enrolment in the PrEP cohort at the KEMRI Research Clinic.

### Qualitative interviews

All individuals newly diagnosed with HIV in the intervention period (both trial participants and partners) and seronegative partners who enroll in the PrEP cohort are offered participation in qualitative in-depth interviews. These individuals can opt out of the interviews and still be followed in the ART or PrEP cohort, according to their preference. During the observation period, individuals are not recruited for interviews.

## ART Cohort for Newly Diagnosed Participants

During the intervention period, all trial participants and partner participants with newly diagnosed acute or prevalent HIV infection are offered referral to the KEMRI Research Clinic, with study staff accompanying individuals to the Research Clinic if possible. At this site, these individuals receive counselling about immediate ART initiation and partner notification (see below). Medications are provided at no cost through PEPFAR. Standard Kenyan first-line regimens are used, with monitoring in accordance with Kenya Ministry of Health guidelines, continuing treatment indefinitely.

Newly diagnosed participants enrolled at each KEMRI Research Clinic are followed for 12 months in the ART cohort, with collection of data and specimens for laboratory testing as detailed in Schedule 4 below. Newly diagnosed persons who do not present to the research clinic within 3 days of their diagnosis are contacted for a discussion of linkage to care at our research clinic or elsewhere if non-research options are preferred. Interim visits may occur at any time during ART cohort follow-up. All interim contacts and visits are documented in participants' study records and on applicable case report forms.

All ART cohort participants undergo a repeat computer audio self-interview (CASI)/computer-assisted personal interview (CAPI) and collection of data on linkage, ART status, and partner outcomes at week 6. This 6-week assessment occurs at the research clinic or at another location of the patient’s choice. If a patient does not accept enrolment in the KEMRI ART Cohort, he or she is referred to one of several large ART programs available in Mombasa, Mtwapa, or Kilifi. We contact these individuals at week 6 to repeat the CASI/CAPI and collect data on linkage results, ART status, and partner outcomes.

## Assisted HIV Partner Services

### Observation period participants diagnosed with HIV infection (index cases)

Standard HIV care guidelines in Kenya recommend testing of partners of the index case through passive partner referral. Patients diagnosed with HIV infection during the observation period are encouraged to refer their partners for this testing. Partner outcomes are self-reported by these patients at the 6-week follow-up visit, after which all observation period index patients are offered standard HIV partner notification services (with research staff, instead of index cases, contacting named partners), using procedures summarized below. Partner outcomes among observation period participants who agree to provide names for standard partner notification are documented by study staff as described below. Of note, testing of partners of index patients diagnosed in the observation period does not include the *Xpert® HIV Qual* test, but consists solely of standard HIV rapid tests conducted according to Kenyan National Guidelines.

### Intervention period participants diagnosed with HIV infection (index cases)

During the intervention period, we administer our enhanced HIV partner notification intervention to all consenting index patients on the day of diagnosis, either at the health facility where the case was diagnosed or at the KEMRI Research Clinic, depending on the patient’s preference. Research staff describe the rationale for providing enhanced HIV partner notification services and procedures for having providers immediately notify partners without directly revealing the index patient’s identity. Specifically, staff indicate that index patients who wish to inform partners themselves are allowed up to 3 days to do so, after which research staff notify partners following standardized procedures. In addition, if an index patient requests that a certain partner not be contacted, the research team respects the patient’s wish and only contacts partners for whom the index patient has given permission. Before consenting participants for partner notification services, we assess for a history or risk of intimate partner violence (IPV) and exclude any individual who is determined to be at high risk of IPV, defined as reporting IPV within the last 1 month.

Prior to initiating interviews, research staff explain that all information collected through the HIV partner notification process will be kept confidential, and reminded that staff will not reveal index cases’ identities when contacting their partners. Index patients who accept HIV partner notification services are interviewed using structured interview forms used to obtain partner contacts and track partner outcomes. Research staff use a timeline-follow back instrument to elucidate the number of unique sex partners that participants have had in the past 1 year for prevalent infection and the past 3 months for acute HIV infection (AHI). If any participant reports injection drug use, information about needle-sharing partners are also elicited.

For each identified sex partner, staff collect information including demographic characteristics, contact information, case’s relationship to the partner, case’s sexual behavior with the partner, and case’s knowledge of the partner’s HIV status. All data are collected using structured interview forms and recorded as numerical or categorical outcomes. Index cases are assigned unique identification numbers and their partners are assigned corresponding numbers that include a random prefix (to protect index case confidentiality) and an additional sequential number for each partner, allowing partners to be linked to index cases in our dataset. Enhanced HIV partner notification intervention participants who are assessed as having a moderate risk for IPV, defined as either (1) a history of IPV during their lifetime either from a current or past partner; or (2) fear of IPV if they participate in the study, undergo special monitoring after the partner notification consent visit.

### Partners of index cases who consent to partner notification services

The schedule of procedures for partner testing is detailed in Schedule 3. When notifying partners, research staff counsel the partner about HIV and encourage them to test at the KEMRI Research Clinic. Partners who are successfully contacted in person are given a study card with a unique ID number linked to the index case’s unique ID number to present at the KEMRI Research Clinic. If contact is established by telephone and the partner agrees to come to the VCT site at the KEMRI Research Clinic for HIV-testing, we send an SMS with an ID number linked to the index case’s unique ID number, to be used when the partner presents for HIV testing.

If a partner refuses to participate in the research study, he or she is offered VCT in a non-research context (i.e., without data collection). After consent, the same CASI/CAPI used in the stepped wedge trial is administered, in order to capture demographic and sexual risk behavior data from participating partners. After the CASI/CAPI, the partner then undergo HIV testing, using standard rapid HIV tests (i.e., standard HIV partner notification services) for partners of observation period index patients and using the HIV-1 RNA testing intervention (i.e., enhanced HIV partner notification services) to detect acute and prevalent HIV infection for partners of intervention period index patients.

If a partner refuses to come to the clinic but consents to enrolment and HIV testing at home, field staff conduct rapid HIV testing in the home and note the result. For partners of intervention period index patients, blood collected at the partner’s home is transported to the KEMRI Research Clinic laboratory for the *XPert® HIV Qual* test. Field staff provide these results to intervention period partners within 48 hours and offer further research options (i.e., ART or PrEP cohort enrolment, enhanced HIV partner notification services if HIV-infected) or referral to HIV prevention services or care as indicated.

Partners of observation period index patients who are found to be seropositive are offered referral to the local HIV care facility of their preference and are not eligible for the ART cohort at the KEMRI Research Clinic. Partners who test seronegative in the observation period are also referred to local facilities for follow-up counseling, and are not eligible for the PrEP cohort at the KEMRI Research Clinic.

Partners of index patients newly diagnosed with acute or prevalent HIV in the intervention period are managed as follows:

- Any partner of an intervention period index patient who is newly diagnosed with acute or prevalent HIV infection is invited to the KEMRI Research Clinic for enrolment into the ART cohort or offered referral for HIV care, if ART cohort enrolment is declined.
- All partners of intervention period index patients who are newly diagnosed with acute or prevalent HIV are also offered enhanced HIV partner notification services, and their partners are also offered enhanced HIV partner notification services, enabling us to thoroughly investigate local sexual networks in which recent transmission occurred.

Partners who are uninfected but in an ongoing partnership with any individual newly diagnosed with acute or prevalent HIV infection in the intervention period are offered enrolment in the PrEP cohort described below.

All partners who are newly diagnosed with HIV in either study period are assessed at a 6-week follow-up visit to determine linkage to care, ART uptake and disclosure to partners. At baseline and at the 6-week visit, research staff counsel newly diagnosed individuals to disclose their HIV status to sexual partners and potential treatment supporters, and provide referral to counseling if needed for that purpose.

## PrEP Cohort for Uninfected Partners in Discordant Partnerships

Uninfected partners in discordant partnerships are invited to enroll in the KEMRI Research Clinic PrEP cohort. These individuals and their partners are encouraged to mutually disclose HIV status, in order to avoid potential unintentional disclosures from participating in research at the same research clinic. PrEP is offered as a bridge to virologic suppression by ART in the index patient. PrEP is continued for at least 6 months, or until the HIV-positive partner has achieved virologic suppression. PrEP medications are provided at no cost by Gilead Sciences Inc., which agreed to provide PrEP medication for up to 75 participants in this study. Standard tenofovir/emtricitibine dosing is used, and WHO and Kenyan guidance on PrEP delivery and safety monitoring is being followed.

MEMS® electronic caps are used to monitor PrEP adherence. At each refill for participants with MEMS® cap pill containers, the electronic data on pill bottle openings since the last refill is uploaded to a database. All women who enroll in the PrEP cohort undergo a family planning assessment and are offered contraception with injectable depot medroxyprogesterone acetate or oral contraceptive pills, both of which are available in the KEMRI Research Clinic. In addition, the use of a barrier methods such as condoms is recommended. Patients who enroll in the PrEP cohort are followed for 12 months, with collection of data and specimens for laboratory testing as detailed in Schedule 5. Interim visits may occur at any time during PrEP cohort follow-up. All interim contacts and visits are documented in participants' study records.

If a PrEP cohort participant presents to a scheduled or interim visit with symptoms suggestive of acute HIV-1 infection syndrome, an *Xpert® HIV Qual* test is performed and PrEP is held if the patient is newly HIV infected. Any PrEP cohort participant who seroconverts is offered enrolment in the ART cohort, with 12 months of follow-up, or referred to the HIV care clinic of their choice. If an uninfected partner in a serodiscordant relationship does not accept enrolment into the PrEP cohort, he or she is counseled on how to reduce HIV transmission risk and referred to another clinic for PrEP and regular HIV testing in the event that risk persists.

## Qualitative Interviews

We invite all participants who enroll in the ART or PrEP cohorts and some newly diagnosed individuals and their partners who are eligible but refuse such enrolment to participate in paired or personal in-depth qualitative interviews to gain insights into intervention uptake, including barriers and facilitators to ART or PrEP uptake and adherence in these groups. Those who are willing to have an in-depth interview are given an appointment at the KEMRI Research Clinic within two weeks of their HIV diagnosis and undergo brief follow-up interviews at each quarterly visit during the 12-month follow-up period (if a cohort participant).

Interviews include questions about whether and to whom the interviewee has disclosed his or her status (if HIV-infected), whether the interviewee has had recent unprotected sex, and the interviewee’s experiences and opinions about taking antiretroviral medications to treat or prevent HIV infection. Initial interviews take approximately one hour, and are conducted at a separate appointment. Participants are reimbursed KSh 500 for their time and travel expenses for this interview. Follow-up interviews for cohort participants take approximately 15 minutes and are integrated into regular study visits. No separate reimbursement is provided for these brief follow-up interviews. Interviews are tape-recorded if the participant consents to this; notes are taken by the interviewer at all interviews. Paired interviews are only be conducted if both partners have mutually disclosed status and agree to a join interview.

For newly diagnosed patients or uninfected partners who decline cohort enrolment but consent to an in-depth interview, we conduct a single interview. For those who enroll in the ART or PrEP cohorts, we aim to conduct four in-depth interviews per interviewee. The first one takes place approximately 2 weeks after diagnosis of acute or prevalent HIV infection or confirmation of uninfected status in an ongoing discordant partnership. Subsequent interviews take place at months 3, 6, 9, and 12, coinciding with cohort follow-up visits.

## Data Analysis for Secondary Outcomes

### Linkage to care

During the intervention period, participants with newly diagnosed HIV infection will be offered referral to our research clinic, and enrolled in ongoing HIV care cohorts. We will evaluate the following outcomes for prevalent HIV, acute HIV, and the combined outcome (acute or prevalent HIV infection): (1) proportion successfully linked to care by week 6; (2) proportion initiating ART by week 6 and month 3 following HIV diagnosis; (3) proportion with viral suppression (<1,000 copies/mL) by month 6 and month 12 following ART initiation. All proportions will be calculated with exact binomial confidence limits, assuming independence between individuals. We will compare these outcomes between prevalent HIV and AHI cases using Pearson Chi square or Fisher exact tests, as appropriate. We will also compare these proportions to outcomes in HIV-infected partners using the same methods.

### Partner testing

We will compare the following week 6 outcomes between the observation and intervention periods: (1) number of partners reported; (2) number of partners successfully contacted; (3) number of partners tested; (4) number of partners newly diagnosed with prevalent HIV infection; (5) number of partners newly diagnosed with AHI ; (6) number of HIV-infected partners newly engaged in care; and (7) number of HIV-uninfected partners initiating PrEP. For PrEP cohort participants (intervention period only) we will also assess the proportion initiating PrEP by month 3 following cohort enrollment and the proportion with adherence >80%, measured by self-report at months 3 and 6 following PrEP initiation. The median, inter-quartile range, and range will be presented for each outcome in each period.

Outcomes will also be compared across study periods using Poisson GEE models with a small-sample variance correction [1]. Potential predictors, adjustment for confounding by calendar time, and interaction testing will be as described for the HIV-1 testing analysis above. We will also analyze local sexual network characteristics including the composition of individuals’ personal sexual networks by attributes, relational type, relational timing, and relational duration. Characteristics will be compared for partners of index cases with AHI vs. prevalent HIV and for networks that include or do not include one or more AHI cases, using Pearson Chi square or Fisher exact tests for binary outcomes and non-parametric tests for continuous outcomes. We will also conduct a subset of these comparisons to the sexual networks of HIV-negative respondents provided through their CASI/CAPI data. Point estimates and standard errors for each behavioral parameter will be used to parametrize the modelling efforts described below.

### Participant barriers and facilitators

Audio recordings of the in-depth interviews will be transcribed verbatim; identifying information will be omitted from transcripts. Transcribed interviews will be entered into NVivo, and analysis will aim to identify and categorize the attitudinal, psychosocial, and contextual factors associated with HIV diagnosis, the testing intervention, and ART or PrEP use. Data analysis will be iterative, and include open coding, axial coding, marginal remarks, comparisons, and memo writing. Themes will be analyzed and triangulated, using a grounded theory framework. The next stage of analysis will relate concepts in order to identify factors that can enhance quality of health services and counselling for HIV-infected patients and their partners.

## Modeling and Cost-Effectiveness Analyses

For the evaluation of impact and cost-effectiveness, precision is more important than power, since estimates will be used as ranges for modelling. Assuming 150 partners tested as a result of the HIV partner notification services intervention, we can estimate their HIV prevalence with reasonable precision (binomial confidence limits, 39.8%–56.3%, for 48.0% prevalence); in addition, we will have 80% power to detect a relative risk (RR) of 1.58 or higher for HIV infection in the partners of AHI patients (n=30), compared to partners of prevalent HIV patients (n=120). Assuming 75 new HIV diagnoses from the HIV-1 RNA testing intervention to detect acute and prevalent HIV infection, we can estimate the proportion attaining virologic suppression with reasonable precision (binomial confidence limits, 69.2%–88.3% if 80.0% achieve suppression by month 12); we will have 80% power to detect an RR of 2.84 or higher for failure to suppress among AHI patients (n=15) compared to prevalent HIV patients (n=60).

We will develop a stochastic, network-based model of HIV-1 transmission within the region. The general approach will follow that developed by Goodreau for modelling HIV-1 transmission among men who have sex with men in multiple international settings, including coastal Kenya [2], extended to a two-sex population. In essence, the model will simulate individuals distinguished by the following attributes: age, sex, sex of partners (males, females, both); sex worker status; circumcision status; HIV status; diagnosis status; time since infection; CD4 count; and treatment status, among possible relevant others revealed by our fieldwork. Individuals will be capable of undergoing any or all of the following transitions: enter the sexually active population; depart from the sexually active population; age; become infected; experience symptoms of AHI; become diagnosed; disclose HIV status to primary or secondary partners; change CD4 count; initiate treatment; change level of treatment adherence; cease treatment; experience opportunistic infections; or die.

Network dynamics will be modelled using the temporal exponential random graph model approach [3]; this approach allows for complex dependences among relations to be modelled explicitly, in forms that preserve multiple features from observed data (e.g., proportion of males with concurrent partners, age mixing matrix) simultaneously. A range of partnership types is common in some parts of Eastern Africa, and likely maintains the persistence of transmission at observed levels, and the model will include these types. Our model will also explicitly include men who have sex with men only, women only, or both, given the epidemiological importance of all three populations in this area.

Population-specific birth and death rates, including general mortality and HIV survival trajectories, will be used to parameterize vital dynamics. Data on population structure will be drawn from the 2014 Kenya Demographic and Health Survey [4] and the 2018 Preliminary KENPHIA 2018 [5], or more recent surveys when available. Existing rates of HIV testing, treatment initiation, adherence, and disease progression and morbidity will be estimated from the latest available literature at the time of model-building. Parameters for sexual network modules will be derived from the partner testing and related network analysis. The impact of the HIV-1 RNA testing intervention and the enhanced HIV partner notification services intervention on diagnosis and linkage to care for index cases and their partners will be estimated from study data; our model will then layer these onto the baseline model to estimate potential population-level impact. Sensitivity analyses will be conducted, especially around key parameters of the individual-level intervention impact. Model outcomes will include life-years lived with HIV or clinical AIDS, life-years gained, incident HIV cases averted, and DALYs averted. We will validate the HIV model using available Kenyan surveillance data [4, 5].

The model will be used to predict the potential impact of our testing and HIV partner notification interventions on HIV incidence, disease progression, and mortality, with additional assessment of the value added by enhanced HIV partner notification for newly diagnosed patients with acute HIV infection. Five main scenarios will be modelled:

1. Baseline model (current guidelines and practice): no HIV-1 RNA testing, low use of PITC
2. Testing for acute and prevalent HIV infection with immediate linkage to care (intervention period)
3. Addition of the enhanced HIV partner notification intervention for newly diagnosed prevalent HIV infections (intervention period)
4. Addition of the enhanced HIV partner notification intervention for newly diagnosed acute HIV infections (intervention period)
5. Addition of PrEP provision to eligible partners tested through the enhanced HIV partner notification intervention (intervention period)

We will perform cost-effectiveness analysis from both the governmental (Ministry of Health) and societal perspectives. The governmental perspective includes only direct medical and direct non-medical costs that would be incurred by the Kenyan government if they implemented the intervention. The societal perspective includes all opportunity costs (i.e., medical costs from the governmental perspective plus costs incurred by patients for transportation and upkeep while seeking care, and costs of lost productivity while traveling to seek care, waiting in line, and seeking care). Costs will be divided into costs of the intervention and costs of HIV treatment. Costs of the intervention will be estimated separately for the HIV testing and enhanced HIV partner notification interventions. Direct medical costs of HIV testing will include costs of testing supplies, personnel, transportation, and communication. Costs of testing supplies are a factor of resource use for testing and unit costs obtained from local medical price lists. Costs of personnel are a factor of wages for clinical, laboratory, counselling, and field workers, and the time spent performing different activities. Time spent performing different activities, including partner tracing, will be obtained by conducting a primary time-and-motion surveys for HIV testing. Transportation cost for HIV partner notification services will be estimated using the mean distance travelled and the travel cost per kilometer based on WHO Choice program data for Kenya [6]. Communication cost will be obtained by estimating the number of minutes per call, the mean number of calls, and costs per call-minute. Costs of HIV treatment include the costs of immediate ART during the intervention period, costs of other medications, and the projected lifetime costs of HIV treatment under different HIV testing and partner notification scenarios. Indirect costs will be estimated using data from patient interviews and publically available data. Questions on patient transport costs, transport time, upkeep costs, and wages will be added to data collection forms. These will be combined with data on patient waiting and patient contact time with health workers to estimate the indirect costs. For unemployed individuals, wages will be estimated based on Kenya’s gross domestic product per capita.

We will combine effectiveness estimates from the HIV-1 RNA testing intervention, linkage to care, and enhanced HIV partner notification interventions with the cost estimates of each intervention to calculate cost per HIV-infected person identified and treated, and the incremental cost-effectiveness ratio (ICER) measured as cost per incident HIV case averted, cost per life-year gained, and cost per DALY averted. Disability weights for calculation of DALYs will be obtained from the latest global burden of disease study [7]. Following WHO guidelines, interventions will be judged to be cost-effective if the ICER is <3 times local GDP and very cost-effective if the ICER is <1 times local GDP per DALY averted [8]. Univariate and probabilistic sensitivity analyses will be performed to determine model robustness and the impact of varying different parameters through their plausible ranges on the estimate of cost-effectiveness.

## References

1. Scott JM, deCamp A, Juraska M, Fay MP, Gilbert PB. Finite-sample corrected generalized estimating equation of population average treatment effects in stepped wedge cluster randomized trials. Stat Methods Med Res. 2017;26:583-597. PMID:25267551.
2. Beyrer C, Baral SD, van Griensven F, Goodreau SM, Chariyalertsak S, Wirtz AL*, et al.* Global epidemiology of HIV infection in men who have sex with men. Lancet 2012,380:367-377. PMID:22819660.
3. Krivitsky PN, Handcock MS, Hunter DR, Goodreau SM, Morris M, Carnegie NB*, et al.* Statnet: tergm: Fit, Simulate and Diagnose Models for Network Evolution Based on Exponential-Family Random Graph Models. 2015 (<https://cran.r-project.org/web/packages/tergm/index.html>, accessed May 2, 2020).
4. Kenya National Bureau of Statistics. Kenya Demographic and Health Survey 2014. Nairobi: Government of Kenya; 2014 (<https://dhsprogram.com/pubs/pdf/fr308/fr308.pdf>, accessed May 2, 2020).
5. National AIDS and STI Control Programme (NASCOP), Preliminary KENPHIA 2018 Report. Nairobi: NASCOP; 2020 (<https://www.nascop.or.ke/kenphia-report/>, accessed May 2, 2020).
6. World Health Organization. Cost effectiveness and strategic planning (WHO-CHOICE). Prices of programme cost inputs. Geneva: WHO; (<http://www.who.int/choice/cost-effectiveness/inputs/prices_prog_cost_input/en/>, accessed May 2, 2020).
7. Salomon JA, Vos T, Hogan DR, Gagnon M, Naghavi M, Mokdad A, et al. Common values in assessing health outcomes from disease and injury: disability weights measurement study for the Global Burden of Disease Study 2010. Lancet 2012,380:2129-2143. PMID:23245605.
8. Drummond MF, Jefferson TO. Guidelines for authors and peer reviewers of economic submissions to the BMJ. The BMJ Economic Evaluation Working Party. BMJ 1996,313:275-283. PMID:8704542.

Schedule 1. Observation Period HIV Testing Cohort

| **Procedures** | **Visit 1** | **Visit 2** |
| --- | --- | --- |
| **Visit week** | **Week 0** | **Week 6^a^** |
| **Date of visit** | **X** | **X** |
| Eligibility Screening | X |  |
| Informed Consent (Observation Period) | X |  |
| Contact Information | X |  |
| Sociodemographic Questionnaire | X |  |
| Risk Assessment Questionnaire | X |  |
| HIV Counseling and Testing^b^ | ± |  |
| Assessment of Linkage to Care, ART, Disclosure, and Self-report of Partner HIV Status^c^ |  | X |
| Offer Standard Assisted Partner Services if Eligible^c^ |  | X |

1. Week 6 procedures are conducted for HIV-positive participants only.
2. HIV testing is performed at the discretion of the health facility provider, using standard rapid antibody tests to confirm prevalent (i.e., seropositive HIV status) in accordance with current Kenyan Ministry of Health testing guidelines.
3. Standard HIV partner notification uses standard rapid antibody tests to confirm prevalent (i.e., seropositive HIV status) in accordance with current Kenyan Ministry of Health testing guidelines

Schedule 2. Intervention Period Point of Care (POC) HIV-1 RNA Testing Cohort

| **Procedures** | **Visit 1** | **Visit 2** |
| --- | --- | --- |
| **Visit week** | **Week 0** | **Week 6^a^** |
| **Date of visit** |  |  |
| Eligibility Screening | X |  |
| Informed Consent (Intervention Period) | X |  |
| Contact Information | X |  |
| Sociodemographic Questionnaire | X |  |
| Risk Assessment Questionnaire | X |  |
| HIV Counseling and Testing^b^ | X |  |
| Intimate Partner Violence Assessment^c^ | X |  |
| Offer Enhanced Assisted Partner Services if Eligible^c^ | X |  |
| Offer ART Cohort Enrolment^c^ | X |  |
| Sample storage | X |  |
| Assessment of Linkage to Care, ART, Disclosure, and Self-Report of Partner HIV Status^c^ |  | X |
| Total Blood Volume per Visit (mL) | 4 |  |

1. Week 6 procedures are conducted for HIV-infected participants only.
2. HIV testing is performed with X-pert testing, followed with rapid antibody tests in RNA positive samples to confirm prevalent (i.e., seropositive HIV status).
3. Only for HIV-infected patients (both AHI and prevalent cases). Enhanced HIV partner notification includes POC X-pert HIV-1 RNA testing, followed with rapid antibody tests in RNA positive samples to confirm prevalent (i.e., seropositive HIV status).

Schedule 3. Partner Testing in the Observation and Intervention Periods

| **Procedures** | **Visit 1** | **Visit 2** |
| --- | --- | --- |
| **Visit week** | **Week 0** | **Week 6^a^** |
| **Date of visit** |  |  |
| Confirmation of partner’s identity | X |  |
| Informed Consent for Partners | X |  |
| Contact Information | X |  |
| Sociodemographic Questionnaire | X |  |
| Risk Assessment Questionnaire | X |  |
| HIV Counseling and Testing^b^ | X |  |
| Intimate Partner Violence Assessment^c^ | X |  |
| Offer Assisted Partner Services if Eligible^c^ | X |  |
| Offer ART Cohort Enrolment if HIV Positive^d^ | X |  |
| Offer PrEP Cohort Enrolment if HIV Negative^d^ | X |  |
| Sample storage^d^ | X |  |
| Assessment of Linkage to Care, ART, Disclosure, and Self-Report of Partner HIV Status^c^ |  | X |
| Total Blood Volume per Visit (mL) | 4 |  |

1. Week 6 procedures are conducted for HIV-infected partners only.
2. In the observation period, HIV testing is performed with standard rapid antibody

tests to confirm prevalent (i.e., seropositive HIV status). In the intervention period, HIV

testing is performed with standard rapid antibody tests when tested at home or

another private location, and with X-pert testing, followed with rapid antibody tests in

RNA positive samples to confirm prevalent (i.e., seropositive HIV status) when tested

at the research clinic.

1. Only for HIV-infected partners in the intervention period
2. Only for partners of patients in the intervention period

Schedule 4. Immediate ART Cohort

| **Procedures at Each Visit** | **V1** | **V2** | **V3** | **V4** | **V5** | **V6** | **V7** | **V8** | **V9** | **V10** | **V11** | **V12** | **V13** | **V14** | **V15** |
| --- | --- | --- | --- | --- | --- | --- | --- | --- | --- | --- | --- | --- | --- | --- | --- |
| **Visit Month**  **(Study Month = 28 days)** | **M0** | **WK2** | **M1** | **WK6** | **M2** | **M3** | **M4** | **M5** | **M6** | **M7** | **M8** | **M9** | **M10** | **M11** | **M12** |
| **Date of visit** |  |  |  |  |  |  |  |  |  |  |  |  |  |  |  |
| Eligibility Screening | X |  |  |  |  |  |  |  |  |  |  |  |  |  |  |
| Informed Consent ART Cohort | X |  |  |  |  |  |  |  |  |  |  |  |  |  |  |
| Contact Information | X | X | X | X | X | X | X | X | X | X | X | X | X | X | X |
| Sociodemographic Questionnaire | X |  |  |  |  |  |  |  |  |  |  |  |  |  |  |
| Risk Assessment Questionnaire | X |  |  |  |  | X |  |  | X |  |  | X |  |  | X |
| HIV Counseling and Testing^a^ | X | X | X |  | X | X |  |  | X |  |  |  |  |  |  |
| Intimate Partner Violence Assessment | X |  |  | X |  | X |  |  | X |  |  | X |  |  | X |
| Risk Reduction Counselling | X | X | X | X | X | X | X | X | X | X | X | X | X | X | X |
| Mental Health Assessment | X |  |  |  |  |  |  |  |  |  |  |  |  |  | X |
| HIV-Positive Cohort Enrolment Questionnaire | X |  |  |  |  |  |  |  |  |  |  |  |  |  |  |
| Medical History | X |  |  |  |  | X |  |  | X |  |  | X |  |  | X |
| Physical Exam (including collection of vaginal and/or rectal swabs as indicated) | X |  |  |  |  | X |  |  | X |  |  | X |  |  | X |
| Hepatitis B Vaccination^b^ | X |  | X |  |  |  |  |  | X |  |  |  |  |  |  |
| Qualitative Interview (if consented for this procedure) |  | (X) |  |  |  | (X) |  |  | (X) |  |  | (X) |  |  | (X) |
| ART Initiation and Refills | X | X | X |  | X | X | X | X | X | X | X | X | X | X | X |
| ART Adherence Counselling | X | X | X | X | X | X | X | X | X | X | X | X | X | X | X |
| ART Adherence Assessment |  | X | X |  | X | X | X | X | X | X | X | X | X | X | X |
| Co-trimoxazole Counseling and Refills | X |  | X |  | X | X | X | X | X | X | X | X | X | X | X |
| TB Assessment, Isoniazid Counseling and Preventive Therapy if Eligible^c^ | X | X | X | X | X | X | X | X | X | X | X | X | X | X | X |
| Urine Pregnancy Test (women only)^d^ | X | (X) | (X) | (X) | (X) | (X) | (X) | (X) | (X) | (X) | (X) | (X) | (X) | (X) | (X) |
| Urinalysis^e^ | X |  |  |  |  |  |  |  |  |  |  |  |  |  | X |
| Urine Collection for Sexually Transmitted Infection Testing (men only) | X |  |  |  |  | X |  |  | X |  |  | X |  |  | X |
| Hepatitis B Surface Antigen | X |  |  |  |  |  |  |  |  |  |  |  |  |  |  |
| Hepatitis C Antibody^f^ | X |  |  |  |  |  |  |  |  |  |  |  |  |  |  |
| RPR with TPHA if Positive | X |  |  |  |  |  |  |  |  |  |  |  |  |  | X |
| Cryptococcal Antigen^g^ | X |  |  |  |  |  |  |  |  |  |  |  |  |  |  |
| Complete Blood Count^e^ | X |  |  |  |  |  |  |  |  |  |  |  |  |  |  |
| Creatinine^e^ | X |  |  |  |  |  |  |  |  |  |  |  |  |  | X |
| Glucose^e^ | X |  |  |  |  |  |  |  |  |  |  |  |  |  | X |
| Cholesterol^e^ | X |  |  |  |  |  |  |  |  |  |  |  |  |  | X |
| ALT^e^ | X |  |  |  |  |  |  |  |  |  |  |  |  |  |  |
| CD4 Count | X |  |  |  |  |  |  |  | X |  |  |  |  |  | X |
| Viral Load | X |  |  |  |  |  |  |  | X |  |  |  |  |  | X |
| Sample Storage | X |  |  |  |  |  |  |  | X |  |  |  |  |  | X |
| Total Blood Volume per Visit (mL) | 20 | 0 | 0 | 0 | 0 | 0 | 0 | 0 | 10 | 0 | 0 | 0 | 0 | 0 | 20 |

1. HIV testing is performed with standard rapid antibody tests baseline to confirm prevalent (i.e., seropositive HIV status). For AHI cases, standard rapid antibody tests are performed at baseline, week 2, month 1, month 2, month 3, and month 6 as needed, to document seroconversion.
2. Hepatitis B vaccination (with doses at baseline, month 1, and month 6) is provided if at high risk for hepatitis B transmission (i.e., female sex workers, men who have sex with men, or male sex workers), as evaluated by the Risk Assessment Questionnaire administered at the baseline and follow-up visits.
3. Isoniazid preventive therapy is considered for ART-naïve individuals who defer ART and for persons taking ART for at least 3 months, in order to avoid potential difficulties with medication adherence and adverse drug effects. Assessment for active tuberculosis is conducted at every cohort visit, as per Kenya National Guidelines.
4. Pregnancy testing is performed at baseline and as clinically indicated thereafter, as per Kenya National Guidelines.
5. Per Kenya National guidelines, we assess hemoglobin (as a complete blood count), creatinine, glucose, lipids, and a urinalysis at baseline. ALT is not be ordered at baseline unless there is a specific indication, such as a history of hepatitis, signs or symptoms of liver disease, or risk of liver disease due to alcohol abuse, chronic hepatitis B or C infection, or concomitant hepatotoxic drug use. Safety monitoring labs with abnormal values at baseline are repeated if clinically indicated.
6. If injection drug use history, as per Kenya National Guidelines.
7. If CD4 count <100 cells/µL.

Schedule 5. PrEP cohort

| **Procedures at Each Visit** | **V1** | **V2** | **V3** | **V4** | **V5** | **V6** | **V7** | **V8** | **V9** | **V10** | **V11** | **V12** | **V13** | **V14** |
| --- | --- | --- | --- | --- | --- | --- | --- | --- | --- | --- | --- | --- | --- | --- |
| **Visit Month**  **(Study Month = 28 days)** | **M0** | **WK2** | **M1** | **M2** | **M3** | **M4** | **M5** | **M6** | **M7** | **M8** | **M9** | **M10** | **M11** | **M12** |
| **Date of visit** |  |  |  |  |  |  |  |  |  |  |  |  |  |  |
| Eligibility Screening | X |  |  |  |  |  |  |  |  |  |  |  |  |  |
| Informed Consent PrEP Cohort | X |  |  |  |  |  |  |  |  |  |  |  |  |  |
| Contact Information | X | X | X | X | X | X | X | X | X | X | X | X | X | X |
| Sociodemographic Questionnaire | X |  |  |  |  |  |  |  |  |  |  |  |  |  |
| Risk Assessment Questionnaire | X |  |  |  | X |  |  | X |  |  | X |  |  | X |
| HIV Counseling and Testing^a^ | X |  | X |  | X |  |  | X |  |  | X |  |  | X |
| Intimate Partner Violence Assessment | X |  |  |  | X |  |  | X |  |  | X |  |  | X |
| Risk Reduction Counselling | X | X | X | X | X | X | X | X | X | X | X | X | X | X |
| Mental Health Assessment | X |  |  |  |  |  |  |  |  |  |  |  |  | X |
| Medical History | X |  |  |  | X |  |  | X |  |  | X |  |  | X |
| Physical Exam (including collection of vaginal and/or rectal swabs as indicated) | X |  |  |  | X |  |  | X |  |  | X |  |  | X |
| Hepatitis B Vaccination^b^ | X |  | X |  |  |  |  | X |  |  |  |  |  |  |
| Qualitative Interview (if consented for this procedure) |  | X |  |  | X |  |  | X |  |  | X |  |  | X |
| PrEP Initiation and Refills^c^ |  | X | X | X | X | X | X | X | X | X | X | X | X | X |
| PrEP Adherence Counselling^c^ | X | X | X | X | X | X | X | X | X | X | X | X | X | X |
| PrEP Adherence Evaluation^c^ |  | X | X | X | X | X | X | X | X | X | X | X | X | X |
| Urine Pregnancy Test (women only)^d^ | X | X | X | X | X | X | X | X | X | X | X | X | X | X |
| Urinalysis | X |  |  |  |  |  |  |  |  |  |  |  |  | X |
| Urine Collection for Sexually Transmitted Infection Testing (men only) | X |  |  |  | X |  |  | X |  |  | X |  |  | X |
| Hepatitis B Surface Antigen | X |  |  |  |  |  |  |  |  |  |  |  |  |  |
| RPR with TPHA if Positive | X |  |  |  |  |  |  |  |  |  |  |  |  | X |
| Creatinine | X |  | X |  | X |  |  | X |  |  |  |  |  | X |
| Sample Storage | X |  | X |  | X |  |  | X |  |  | X |  |  | X |
| Total Blood Volume per Visit (mL) | 10 | 0 | 4 | 0 | 8 | 0 | 0 | 8 | 0 | 0 | 8 | 0 | 0 | 10 |

1. HIV testing is performed with standard rapid antibody tests at all visits. At baseline, HIV testing includes *X-pert® HIV Qual* testing to test for AHI.
2. Hepatitis B vaccination (with doses at baseline, month 1, and month 6) is provided if at high risk for hepatitis B transmission (i.e., female sex workers, men who have sex with men, or male sex workers), as evaluated by the Risk Assessment Questionnaire administered at the baseline and follow-up visits.
3. PrEP is offered after review of baseline test results (creatinine, HBsAg) to all participants in this seronegative cohort. PrEP is continued until the seropositive partner has achieved virologic suppression on ART or until the partnership has dissolved, provided the participant does not have a new HIV-infected partner. PrEP counseling is offered at each visit regardless of uptake. After PrEP initiation, each visit includes adherence counselling, adherence evaluation, and refills until PrEP is discontinued. A MEMS® electronic cap is issued to each PrEP cohort participant in order to monitor adherence.
4. Pregnancy testing is provided at every visit. PrEP is discontinued for any woman who becomes pregnant in the study. Such individuals are provided HIV risk reduction counseling and referred for antenatal care.
