# Supplementary material for: A Novel HIV-1 RNA Testing Intervention to Detect Acute and Prevalent HIV Infection in Young Adults and Reduce HIV Transmission in Kenya: Protocol for a Randomized Controlled Trial
Source: JMIR Res Protoc. 2020 Aug 7;9(8):e16198. doi: 10.2196/16198 (PMC7442943; doi:10.2196/16198)
Supplement: Multimedia Appendix 2 [file resprot_v9i8e16198_app2.docx]

# Multimedia Appendix 2: *Tambua Mapema Plus* Eligibility Form

**Start time: ______________ End time: _______________** Volunteer ID: _________

**Screening ID:** *5-digits auto generated* **Facility name:** _____________________

Date: ____/ ____/ ____

Gender: ____ Male ___ Female

Estimated age: ____ (years)

*Permission script: We are currently conducting a study in this facility for which only some patients are eligible. We don’t need your name or contact information in order to see if you’re eligible. Is it OK if I ask a few questions to see if you are eligible? This will take about one minute.*

Research staff initials documenting verbal permission to screen: _________________

Axillary temperature: **_______**

DOB: (dd/mmm/yyyy): ____/ ____/ ____ Actual age: ____ (years)

*(For participants aged 18 and 39, please calculate the exact age in years and months and document in the comments section on the flip side of the form)*

Ever tested for HIV? __Yes __ No

HIV Status: __ Positive __ Negative/ Unknown

If HIV positive: registered in care: __Yes __ No

If HIV positive: on ART: __Yes __ No

**Patients not in care or on ART should be counselled accordingly.**

Have you previously enrolled in this study? __Yes __ No

If yes, which facility: _______________________________ Date: (dd/mmm/yyyy): ____/ ____/ ____

**(Do not proceed with screening and enrolment if the patient has been enrolled in TMP within 6 months at any of the TMP study sites)**

**If between 18 and 39 years of age and not known HIV positive, calculate risk score below:**

| **Characteristic** | **Present = Yes (circle score)** | **Absent = No (circle score)** |
| --- | --- | --- |
| Age **18-29 years** | 1 | 0 |
| Reported **fever** | 1 | 0 |
| Reported **diarrhoea** | 1 | 0 |
| Reported **fatigue** | 1 | 0 |
| Reported **body aches** | 1 | 0 |
| Reported **sore throat** | 1 | 0 |
| Reported **genital ulcer** | 3 | 0 |
| TOTAL SCORE |  |  |

**If score is 2 or more (≥ 2), invite patient for Tambua Mapema Plus study.**

Research staff Initials: _________________

**If enrolled, place sticker with participant ID number in box in right hand corner above. Please confirm all screening data above after written consent is obtained:**

Research staff Initials: _________________

**Please capture reason not enrolled in the table below.**

| **Reason** | **Check all applicable** |
| --- | --- |
| Not eligible |  |
| Too ill |  |
| Too busy, in a hurry |  |
| Need to ask partner |  |
| Temporary visitor (<2 weeks in study area) |  |
| Outside catchment area (>30 km from the KEMRI Mtwapa or Kilifi Research Clinic) |  |
| Never been sexually active |  |
| Doesn’t want to participate in research |  |
| Patient enrolled in TMP study within 6 months at any of the TMP study sites |  |
| Patient found to be under or over age [correct age and initial above] |  |
| Patient found to have known HIV-positive status [correct and initial above] |  |
| Patient does not want to take a HIV test |  |
| Other, describe: ________________________ |  |

Comments: ___________________________________________________________________
