# Supplementary material for: A Novel HIV-1 RNA Testing Intervention to Detect Acute and Prevalent HIV Infection in Young Adults and Reduce HIV Transmission in Kenya: Protocol for a Randomized Controlled Trial
Source: JMIR Res Protoc. 2020 Aug 7;9(8):e16198. doi: 10.2196/16198 (PMC7442943; doi:10.2196/16198)
Supplement: Multimedia Appendix 3 [file resprot_v9i8e16198_app3.docx]

| Multimedia Appendix 3: Computer-Assisted Self-Interview/Computer-Assisted Personal Interview *Start time: ___ ___ : ___ ___ hrs* Participant ID _______ |
| --- |
| *We would like to get some information about your sexual behaviour as part of this study. There are many kinds of sex. For this questionnaire, we are interested in two kinds: sex when a man’s penis goes inside a woman’s vagina (vaginal sex) or anus (anal sex), or sex when a man’s penis goes inside a man’s anus (anal sex).*  *Please answer to the best of your ability.* |
| 1. When did you last have sexual intercourse? (Circle one)   *1=Within past 2 days 2=Within past week 3=Within past 2 weeks 4= Within past 6 weeks*  *5=More than 6 weeks ago 6=Never had sex* |
| If Q1 response = 5 or 6, then skip to Q50. |
| 1. How many people have you had sexual intercourse with in the last 6 weeks? If you are not sure of the exact number, please take your best guess. _____ |
| 1. Of these people, how many were women? _____ |
| 1. Of these people, how many were men? _____ |
| 1. Of the people you had sex with in the last 6 weeks, how many were in each of the following categories:   *Spouse _____ Regular (long-term partner other than spouse) _____*  *Casual (short-term partner) _____ One-time encounter _____* |
| 1. Of the people you had sex with in the last 6 weeks, how many were in each of the following categories:   *They paid you money, gifts or your living expenses for sex _____*  *You paid them money, gifts or their living expenses for sex _____* |
| *I am now going to ask about the person you had sex with most recently in the past 6 weeks.* |
| 1. Was your most recent partner a man or a woman?   *Man Woman* |
| 1. How would you describe your relationship with this person?   *1=Spouse 2=Regular partner 3=Casual partner 4=One-time encounter*  *5=Other: ______________ (type description)* |
| 1. When did you last have sex with this person? If you do not know the exact date, please make your best guess.   Date: ___ / ___ / _____ (day / month / year) (If Q8=4, skip to Q12) |
| 1. When did you have sex with this person for the very first time? If you do not know the exact date, please make your best guess.   Date: ___ / ___ / _____ (day / month / year) |
| 1. Do you expect to have sex with this person again in the future? (Circle one)   *1=Definitely yes 2=Probably yes 3=Not sure 4=Probably no*  *5=Definitely no* |
| 1. What is the age of this person? (Circle one)   *1=About the same age as me 2=More than 5 years older than me 3=More than 5 years younger than me* |
| 1. If Q8 response is not 1: Is he or she married? *Yes No Don’t know* |
| 1. As far as you know, has this person had any other sexual partners besides you in the last 6 weeks? (Circle one)   *1=Definitely yes 2=Probably yes 3=Not sure 4=Probably no 5=Definitely no* |
| 1. If Q14 response ≤2: As far as you know, are this person’s other partners male or female? (Circle one)   *1=Male 2=Female 3=Both 4=Not sure* |
| 1. When you last had sex with this person, did you use a condom? *Yes No Don’t remember* |
| 1. Heterosexual partnerships: If volunteer is male (Form 2A Staff Entered) and current partner is female (Q7 = woman) or if volunteer is female (Form 2A Staff Entered) and current partner is male (Q7 = man):   17a. During your relationship so far, how often have you had vaginal sex with this partner on average? (Circle one)  *1=Daily 2=Almost every day 3=Once a week 4=Less often than once a week*  17b. When you have had vaginal sex with this partner, how often did you use a condom?  *1=Always 2=Most of the time 3=About half the time 4=Rarely 5=Never*  17c. During your relationship so far, how often have you had anal sex with this partner on average? (Circle one)  *1=Daily 2=Almost every day 3=Once a week 4=Less often than once a week 5=Never*  17d. If Q17c response is not never: When you have had anal sex with this partner, how often did you use a condom?  *1=Always 2=Most of the time 3=About half the time 4=Rarely 5=Never* |
| 1. MSM: If volunteer is male (Form 2A Staff Entered) and current partner is male (Q7 = man):   18a. During your relationship so far, how often have you had anal sex with this partner in an average week? (Circle one)  *1=Daily 2=Almost every day 3=Once a week 4=Less than once a week 5=Never*  18b. If Q18a response is not never: When you have had anal sex with this partner, how often did you use a condom?  *1=Always 2=Most of the time 3=About half the time 4=Rarely 5=Never*  18c. When you have anal sex with this partner, what role do you take? (Circle one)  *1=Exclusively insertive 2=Mostly insertive*  *3=Insertive about half the time and receptive about half the time*  *4=Mostly receptive 5=Exclusively receptive* |
| 1. What do you think this person’s HIV status is? (Circle one)   *1= HIV positive 2= Probably HIV positive 3=I have no idea 4= Probably HIV negative*  *5= HIV negative* |
| 1. If Q19 response is not 3: Please indicate all of the reasons you have for thinking this (circle all that apply)   *1=We discussed our HIV status 2=We did couple counselling together*  *3=He / she seemed healthy 4= He /she seemed unhealthy 5= I heard rumours*  *6=He / she told me is taking ARVs 7=I have seen him or her taking pills 8=Don’t know* |
| If Q2>1, continue for the second most recent partner. Otherwise, skip to Q50. |
| *I am now going to ask about the person you had sex with the second most recently in the past 6 weeks.* |
| 1. Your second most recent partner, was this person a man or a woman? *Man Woman* |
| 1. How would you describe your relationship with this person?   *1=Spouse 2=Regular (long-term) partner 3=Casual (short-term) partner*  *4=One-time encounter 5=Other: ______________ (type description)* |
| 1. When did you last have sex with this person? If you do not know the exact date, please make your best guess.   Date: ___ / ___ / _____ (day / month / year) (If Q22=4, skip to Q26) |
| 1. When did you have sex with this person for the very first time? If you do not know the exact date, please make your best guess.   Date: ___ / ___ / _____ (day / month / year) |
| 1. Do you expect to have sex with this person again in the future? (Circle one)   *1=Definitely yes 2=Probably yes 3=Not sure 4=Probably no*  *5=Definitely no* |
| 1. What is the age of this person? (Circle one)   *1=About same age as me 2=More than 5 years older than me 3=Less than 5 years younger than me* |
| 1. If Q22 response is not 1: Is he or she married? *Yes No Don’t know* |
| 1. As far as you know, has this person had any other sexual partners besides you in the last 6 weeks? (Circle one)   *1=Definitely yes 2=Probably yes 3=Not sure 4=Probably no 5=Definitely no* |
| 1. If Q28 response ≤2: As far as you know, are this person’s other partners male or female? (Circle one)   *1=Male 2=Female 3=Both 4=Not sure* |
| 1. When you last had sex with this person, did you use a condom? *Yes No Don’t remember* |
| 1. Heterosexual partnerships: If volunteer is male (Form 2A Staff Entered) and current partner is female (Q21 = woman) or if volunteer is female (Form 2A Staff Entered) and current partner is male (Q21 = man):   31a. During your relationship so far, how often have you had vaginal sex with this partner on average? (Circle one)  *1=Daily 2=Almost every day 3=Once a week 4=Less often than once a week*  31b. When you have had vaginal sex with this partner, how often did you use a condom?  *1=Always 2=Most of the time 3=About half the time 4=Rarely 5=Never*  31c. During your relationship so far, how often have you had anal sex with this partner on average? (Circle one)  *1=Daily 2=Almost every day 3=Once a week 4=Less often than once a week 5=Never*  31d. If Q31c response is not never: When you have had anal sex with this partner, how often did you use a condom?  *1=Always 2=Most of the time 3=About half the time 4=Rarely 5=Never* |
| 1. MSM: If volunteer is male (Form 2A Staff Entered) and current partner is male (Q21 = man):   32a. During your relationship so far, how often have you had anal sex with this partner in an average week? (Circle one)  *1=Daily 2=Almost every day 3=Once a week 4=Less than once a week 5=Never*  32b. If Q32a response is not never: When you have had anal sex with this partner, how often did you use a condom?  *1=Always 2=Most of the time 3=About half the time 4=Rarely 5=Never*  32c. When you have anal sex with this partner, what role do you take? (Circle one)  *1=Exclusively insertive 2=Mostly insertive*  *3=Insertive about half the time and receptive about half the time*  *4=Mostly receptive 5=Exclusively receptive* |
| 1. What do you think this person’s HIV status is? (Circle one)   *1= HIV positive 2= Probably HIV positive 3=I have no idea 4= Probably HIV negative*  *5= HIV negative* |
| 1. If Q33 response is not 3: Please indicate all of the reasons you have for thinking this (circle all that apply)   *1=We discussed our HIV status 2=Did couple counselling together*  *3=He / she seemed healthy 4= He /she seemed unhealthy 5= I heard rumours*  *6=He / she told me is taking ARVs 7=I have seen him or her taking pills 8=Don’t know* |
| If Q2>2, continue for the third most recent partner. Otherwise, skip to Q49. |
| *I am now going to ask about the person you had sex with the third most recently in the past 6 weeks.* |
| 1. Your third most recent partner, was this person a man or a woman? *Man Woman* |
| 1. How would you describe your relationship with this person?   *1=Spouse 2=Regular (long-term) partner 3=Casual (short-term) partner*  *4=One-time encounter 5=Other: ______________ (type description)* |
| 1. When did you last have sex with this person? If you do not know the exact date, please make your best guess.   Date: ___ / ___ / _____ (day / month / year) (If Q36=4, skip to Q40) |
| 1. When did you have sex with this person for the very first time? If you do not know the exact date, please make your best guess.   Date: ___ / ___ / _____ (day / month / year) |
| 1. Do you expect to have sex with this person again in the future? (Circle one)   *1=Definitely yes 2=Probably yes 3=Not sure 4=Probably no*  *5=Definitely no* |
| 1. What is the age of this person? (Circle one)   *1=About same age as me 2=More than 5 years older than me 3=Less than 5 years younger than me* |
| 1. If Q36 response is not 1: Is he or she married? *Yes No Don’t know* |
| 1. As far as you know, has this person had any other sexual partners besides you in the last 6 weeks? (Circle one)   *1=Definitely yes 2=Probably yes 3=Not sure 4=Probably no 5=Definitely no* |
| 1. If Q42 response ≤2: As far as you know, are this person’s other partners male or female? (Circle one)   *1=Male 2=Female 3=Both 4=Not sure* |
| 1. When you last had sex with this person, did you use a condom? *Yes No Don’t remember* |
| 1. Heterosexual partnerships: If volunteer is male (Form 2A Staff Entered) and current partner is female (Q35 = woman) or if volunteer is female (Form 2A Staff Entered) and current partner is male (Q35 = man):   45a. During your relationship so far, how often have you had vaginal sex with this partner on average? (Circle one)  *1=Daily 2=Almost every day 3=Once a week 4=Less often than once a week*  45b. When you have had vaginal sex with this partner, how often did you use a condom?  *1=Always 2=Most of the time 3=About half the time 4=Rarely 5=Never*  45c. During your relationship so far, how often have you had anal sex with this partner on average? (Circle one)  *1=Daily 2=Almost every day 3=Once a week 4=Less often than once a week 5=Never*  45d. If Q45c response is not never: When you have had anal sex with this partner, how often did you use a condom?  *1=Always 2=Most of the time 3=About half the time 4=Rarely 5=Never* |
| 1. MSM: If volunteer is male (Form 2A Staff Entered) and current partner is male (Q35 = man):   46a. During your relationship so far, how often have you had anal sex with this partner in an average week? (Circle one)  *1=Daily 2=Almost every day 3=Once a week 4=Less than once a week 5=Never*  46b. If Q46a response is not never: When you have had anal sex with this partner, how often did you use a condom?  *1=Always 2=Most of the time 3=About half the time 4=Rarely 5=Never*  46c. When you have anal sex with this partner, what role do you take? (Circle one)  *1=Exclusively insertive 2=Mostly insertive*  *3=Insertive about half the time and receptive about half the time*  *4=Mostly receptive 5=Exclusively receptive* |
| 1. What do you think this person’s HIV status is? (Circle one)   *1= HIV positive 2= Probably HIV positive 3=I have no idea 4= Probably HIV negative*  *5= HIV negative* |
| 1. If Q47 response is not 3: Please indicate all of the reasons you have for thinking this (circle all that apply)   *1=We discussed our HIV status 2=Did couple counselling together*  *3=He / she seemed healthy 4= He /she seemed unhealthy 5= I heard rumours*  *6=He / she told me is taking ARVs 7=I have seen him or her taking pills 8=Don’t know*   1. Have you participated in group sex (that is sex with more than 1 person at the same time) in the past 6 weeks? *1=Yes 2=No*  \| ***IPV Questions: “I would like to ask you some questions about your current and past relationships.*** \| \| --- \|  1. Have you ever been in a relationship with a person who physically hurt you?   *1=Yes 2=No* (If Q50=No, skip to Q51)  How recently did this happen?  *1=In the past 1 month 2=Not in the past 1 month*   1. Have you ever been in a relationship with a person who threatened, frightened, or insulted, or treated you badly?   *1=Yes 2=No* (If Q51=No, skip to Q52)  How recently did this happen?  *1=In the past 1 month 2=Not in the past 1 month*   1. Have you ever been in a relationship with a person who forced you to participate in sexual activities that made you feel uncomfortable?   *1=Yes 2=No* (If Q52=No, skip to Q53)  How recently did this happen?  *1=In the past 1 month 2=Not in the past 1 month* |
| \| ***Injection Drug Use Questions: “I would like to ask you some questions about whether you inject drugs such as heroin, cocaine, or other drugs.”*** \| \| --- \| |
| 1. Have you injected drugs in the past 6 weeks? *1=Yes 2=No* |
| 1. If Q53 = yes: Did you share needles with someone else at any time in the past 6 weeks? 1=*Yes 2=No* |
| 1. If Q53 = yes: How often do you inject drugs? *1=Daily 2=Less than daily* |

Stop time: ___ ___ : ___ ___ (hours)

***This* *concludes our questionnaire. Thank you very much for your time. The study team will be in touch with you again after your clinic visit and any testing ordered have been completed.***
