# Supplementary material for: A Novel HIV-1 RNA Testing Intervention to Detect Acute and Prevalent HIV Infection in Young Adults and Reduce HIV Transmission in Kenya: Protocol for a Randomized Controlled Trial
Source: JMIR Res Protoc. 2020 Aug 7;9(8):e16198. doi: 10.2196/16198 (PMC7442943; doi:10.2196/16198)
Supplement: Multimedia Appendix 4 [file resprot_v9i8e16198_app4.docx]

# **Multimedia Appendix 4: Focus Group Topic Guides**

| **Focus group discussion topic guide for participating health facility staff**  **Pre-Intervention Topic Guide** |
| --- |

Study number: _________________________________

Interviewer : _________________________________

Site : _________________________________

Date : _________________________________

Start : _________________________________

End : _________________________________

The objectives of the discussions are to assess and explore:

Healthcare providers’ experiences with HIV testing and diagnosis, care and treatment in existing healthcare system

Healthcare providers’ knowledge base on HIV infection and acute HIV, and identify training needs

How partner notification and testing is currently performed, and identify opportunities for implementing assisted partner services intervention (aPS)

Healthcare providers’ perspectives on potential barriers and facilitators for malaria testing and PITC

**Introduction:**

In this interview/discussion we want to understand your approach to patients who present for urgent care with febrile illness like symptoms. We would like to understand your differential diagnosis, what prompts a healthcare worker to test for HIV versus thinking of other pathogens such as Malaria, bacterial infections etc.

In this interview/discussion we would also want to understand your HIV testing services at your health facility and ask you as healthcare workers for your views on early detection of HIV infection and prevention of transmission through finding and testing the partners of newly diagnosed individuals. We would also appreciate to learn from you what you know about acute HIV infection, and whether you feel screening for acute HIV infection is important, feasible and scalable.

With your permission, we would like to tape record the interview/discussion for later transcription and translation to English for analysis. All information will be kept strictly confidential, and, as participants, you will never be identified with any comment you make.

1. **Health facility background**

- How would you describe the patients that are attended to at this health facility?

(*Probe for: Insurance covered; socio-economic status; nature of illnesses; antenatal/post-natal services*)

- Kindly, describe the type of HIV testing that you offer at your health facility.

(*Probe for the types and numbers of health* providers *that are in the clinic, services offered; what guides the particular services offered*)

- How would you say the HIV services are organised in this health facility?

(probe for: Who supports the HIV services offered in your health facility; government’s role; who are the collaborators and their mandate; If insurance companies including NHIF support HIV services and to what extent; do they have a VCT? CCC?

1. **HIV diagnostic services**

- What avenues for HIV testing are available?

(Pr*obe lab, VCT, ANC etc*.)

- Who initiates HIV testing

*(Prompt: should staff other than clinician also initiate HTC?)*

- What are the Point of care HIV tests available at your health facility?

(*Probe for who uses them, knowledge of other POC tests, knowledge on applications for POC tests*)

- What kind of patients are tested for HIV in this health facility? And why?

(*Probe symptoms/clinical presentation prompts them to refer for HIV testing*)

- Kindly describe for me how provider-initiated testing and counselling (PITC) works in your health facility.

(*Probe on: who is referred to VCT vs the lab; who makes the referrals; at what point counselling occurs; who are the staff involved in testing for HIV*)

- What would you say are some of the reasons why health providers may not offer routine HIV testing?
- Are there occasions when patients are referred for HIV testing but do not test? If yes, why?
- What challenges do you face in providing routine HIV diagnostic services?

(*Probe for stock outs, training challenges, staffing, supplies etc.*)

- Who provides the HIV training services? How frequently? Which staff are sent for the training?
- Generally, what are your opinions about HIV testing services that you offer at your health facility?

*(probe for: perceptions on the extent of testing done; what they would find acceptable testing rate for the health facility; what needs to be considered to improve HIV testing*)

- How does your health facility handle discrepant rapid HIV test results that could potentially be acute HIV?
- Do you know symptoms associated with HIV seroconversion, that is, acute HIV?
- What would you say is the importance of diagnosing acute HIV infection?
- Do you think it is important to include in the current national HIV guidelines screening for acute HIV?
- If there were to laboratory point of care tests available for diagnosing AHI, what factors would you say should be considered? Are there any barriers you foresee?

(*Probe for suggestions on potential point of care tests to target AHI, volume workload, benefits over using reference laboratories, space within laboratories, who would operate the POC tests, training of HCPs, educating the community and at-risk groups*)

1. **Partners notification, finding and testing for HIV**

- How does the health facility ensure that partners of HIV positive patients also test for HIV?

*(Probe for ways in which partner notification is done if any; the points at which it is done; the staff involved and their roles)*

- Who initiates the process of partner testing?
  - Who follows up whether all reported partners have been tested for HIV and linked to care if positive?
- In your experience what would you say about how easy or difficult it is for HIV positive patients to list their sexual partner both current and past sexual partners?
- In your context or based on the kind of patients you attend to at this health facility, what do you think would be preferred way to make sure partner of HIV positive patients also get tested?
- If you were to assist a HIV positive client/patient to contact their partner(s) for disclosure and HIV testing, how would you go about it?
  - What factors would you consider? What would be your fears and concerns?
- Kenya is beginning to implement the provider notification approach or also called ‘assisted partner services’. *Assisted partner services involves index patients tested with HIV infections being asked to provide information about sex partners and then a health provider contacts these partners to ensure that they test for HIV and link to care,* ***without revealing the identity of the index patient****)*
  - Have you heard of assisted partner services (aPS)?
  - Do you have any experience with assisted partner services? If yes, probe for: *who does it and a description of how it is done, the role of the health provider*
  - If aPS not done, how do you think assisted partner services can be done? What would you say is feasible?
- Who should provide aPS?

*(probe for: outreach workers? Clinicians? HTS counsellors?)*

- Would you say aPS as an intervention is feasible? What about scalable?
- When would you say is the appropriate time as a health provider to offer assisted partner services for patients newly diagnosed with HIV?

*(Probe for immediate vs delayed aPS)*

- What challenges do you foresee (or experiencing) in implementing aPS?
- What support would healthcare providers require for the implementation of assisted partner services?

(*Probe for training, guidelines,supervision, experience sharing*)

1. **HIV care at the health facility**

- Describe what you would ordinarily do if you received a newly HIV diagnosed case.
- What would you say are the kind of HIV clinical care and support services that you offer in your clinic?

(*Probe for clinical care, support (e.g. couples counselling, adherence, retention, referral to support groups)*)

- What barriers would you say you experience in providing HIV care and treatment
  - *Do you remember any problematic situation in your work you found particularly challenging when attending to HIV positive patients?*
- How do you monitor HIV treatment?

*(Probe for: Reference labs, Viral Load testing)*

- What would you consider you have done well in regard to HIV testing, care and management?

(*Probe for understanding of UNAIDS 90-90-90 targets and what the facility is doing to contribute to the targets, recommendations for improvement*)

1. **Malaria screening and management**

- How would you describe the patients you test for malaria?
- What is the approach to the febrile patient who presents to your health facility?
- What are some of the symptoms of patients who you refer for malaria testing?
- What malaria tests do you offer in your facility?

*(Probe: what guides them to use blood slide vs mRDT)*

- What do malaria guidelines say about who should be tested and treated for malaria?
- How often do you go for training on managing the acute febrile patient- and malaria guidelines specifically?

*(Probe for who attends the training)*

- What challenges do you face in providing routine malaria diagnostic services?

*(Probe for stock outs, training challenges, staffing, supplies etc.)*

**Other comments and recommendations**

- Any other comments you have regarding the discussion and the *Tambua Mapema Plus* study? What about any recommendations that we may have not discussed?

*Kindly thank the participants for sharing their views.*

**END**

| **Focus group discussion topic guide for participating health facility staff**  **Post-Intervention Topic Guide** |
| --- |

Study number: _________________________________

Interviewer : _________________________________

Site : _________________________________

Date : _________________________________

Start : _________________________________

End : _________________________________

The objectives of the discussions are to assess and explore:

Healthcare providers perspective on the impact of HIV testing during TMP study at health facilities, and barriers and facilitators on the scale up of PITC

Healthcare provides understanding and experience with assisted HIV partner notification services, immediate treatment, and PrEP use as HIV prevention strategies

**Introduction:**

In this interview/discussion we want to understand how you feel about the Tambua Mapema plus study that has been on-going in your facility. We would like to hear from you what impact you think the study had on HIV testing services at the facility, and whether you think the interventions are scalable.

In this interview/discussion we would also be interested in your opinion on assisted HIV partner notification services, its feasibility and any challenges in its implementation at your facility, your experience with immediate or same day ART treatment, and PrEP provision, uptake and retention of your cohorts. In some places we will use data collected during the implementation of the study to guide our discussion.

With your permission, we would like to tape record the interview/discussion for later transcription and translation to English for analysis. All information will be kept strictly confidential, and, as participants, you will never be identified with any comment you make.

1. **Implementation of Tambua Mapema Plus Study**

KEMRI in collaboration with the county ministry of health and individual participating health facilities has been implementing in this health facility a study called “Tambua Mapema Plus”. This study had an intervention phase whose aim was to determine whether HIV can be diagnosed in patients seeking care at health facilities, with immediate referral and care for HIV-infected patients and their partners. Patients seeking care at the health facility, meeting the study enrolment criteria and willing to participate were consented to participate in the study. Individuals were offered qualitative HIV-1 RNA testing with the Cepheid GeneXpert and those who were diagnosed with HIV and accepted were put on immediate treatment and enrolled in an ART cohort at KEMRI. Also, those who consented were offered assisted partner services. Partners who tested positive were also enrolled in the ART cohort, while partners who tested negative in discordant relationships, and were willing, were enrolled in the PrEP cohort.

- What did you think of the Tambua Mapema Plus study?
- What would you say has been the impact of the study in your health facility?
  - On HIV testing services? Has there been an increase in the number of people presenting for care tested for HIV?
  - On ART services? Has there been any increase in patients starting immediate or same day treatment? What has been the facilities experience with retention in care of these participants?
  - What did you think of patients diagnosed in your facility being enrolled in ART cohort at KEMRI? Did this affect your reporting of HIV testing and linkage data to NASCOP/MOH? Were there any other challenges you experienced?
  - What would you say were the challenges experienced in the facility during the implementation of TMP study?
  - In your opinion how would you say your health facility and colleagues / staff benefited from the implementation of the Tambua Mapema Plus study if any?
  - Do you believe that the health facility staff should have played a more active role in the implementation of the study? In what way?
- Do you believe that your health facility was the appropriate one for the implementation of the Tambua Mapema Plus study? Why?
- What factors should the study team have considered during the implementation of the study?
  - Training of health facility staff on their roles in the study – how should it have been done?
  - Raising awareness – how?
- What ideas do you have to help with the improvement of the interventions if they are to be scaled up? i.e. POC HIV RNA testing?

1. **HIV Testing**

*HIV Testing Health Facility Data*

| ***Observation Period*** | | | | ***Intervention Period*** | | | |
| --- | --- | --- | --- | --- | --- | --- | --- |
| *# patients enrolled 18-39* | *Proportion sent for HIV* | *Proportion tested for HIV* | *Proportion found to be HIV positive (Prevalence)* | *# patients enrolled in TMP 18-39* | *Proportion tested for HIV* | *Proportion found to be HIV positive both acute and prevalent HIV (Prevalence)* | *# refused HIV testing* |
|  |  |  |  |  |  |  |  |

- What do you think about the data provided above?
  - Number enrolled verses number sent for HIV testing between observation and intervention periods, what is different? What is likely to be the explanation for the difference if any?
  - What about number of patients testing HIV positive between observation and intervention periods, is it different? what is different? What is likely to be the explanation for the difference?
  - What would you do differently in your HIV testing services at the facility in view of the above results?
  - Would you say there is a likelihood that there are individuals with prevalent or acute HIV infection that are missed while seeking urgent care?
  - What do we need to do to ensure people are diagnosed early?
  - As a healthcare provider, what do you do when you diagnose a patient with HIV?
    - What kind of counselling do you give such a patient?
  - If you got a patient diagnosed with acute HIV infection, how are you likely to handle such a case?
    - What different counselling would you give such a patient?
  - What about patients refusing to be tested for HIV?
    - What would you say are some of the reasons patients give for refusing to take an HIV test? How often do you get refusals?
    - Do you think that the recommendation you give as a provider helps patients accept HIV testing?
    - What are some arguments you have used to try to get patients to test?
  - What criteria do you use to decide who is tested for HIV and who is not?
    - Do you think providers can tell if a patient has HIV? If so, why? If not, why not?
  - What would you say needs to happen to increase diagnosis of HIV infection at your facility after this study?
  - What would you say needs to happen to find the undiagnosed HIV cases in the community?

1. **Partners notification, finding and testing for HIV**

*One of the interventions the Tambua Mapema plus study was implementing is the assisted HIV partner services. Assisted partner services for index patients with HIV infections involves elicitation of information about sex partners and then contacting these partners to ensure that they test for HIV and link to care,* ***without revealing the identity of the index patient.***

***The outcome of the intervention was ……***

Partner Notification Intervention Outcome

| *# of HIV positive patients* | *# of partners reported* | *# of partners tested* | *# of partners testing HIV positive* | *# enrolled into PrEP* |
| --- | --- | --- | --- | --- |
|  |  |  |  |  |

- Have you heard about assisted partner notification?
  - What would you say is involved in implementing aPS?
  - Is your health facility implementing aPS?
  - WHO recommends aPS and recently the Kenyan MOH adopted the approach alongside contract referral and client referral as options of partner notification which are recommended for patients who test HIV positive. Are you aware of these developments?
- Looking at the data drawn from the TMP study on partner notification:
  - what do you think about the differences between the number of HIV positive patients verses the number of partners reported?
  - What about the comparison between the number of partners reported verses number of partners tested? What would explain the difference?
  - Majority of the patients reported one partner when interviewed about partners, how do you think healthcare workers can get information about other partners besides the primary partner from HIV positive patients?
- What is your opinion about partner notification as an intervention for HIV prevention?
- Do you have any experience with any of the partner notification approaches that is assisted partner services, contract referral or client referral?
  - If yes, probe for which one? *who provides and a description of what is actually done, the role of the health provider*
  - *What about couple counselling? and how would couple counselling experience inform assisted partner services*
- Any challenges you foresee (or have experienced) in implementing partner notification?

1. **Immediate ART treatment**

*Patients that were diagnosed with HIV during the intervention phase of the study were enrolled into care and initiated on treatment immediately – same day initiation for most of the participants.*

(include here brief findings in figures of those tested positive and initiated on ART in TMP study)

| *# of HIV positive index patients* | *# of partners testing HIV positive* | *Total # of HIV positive cases* | *# initiated on Treatment* | *# enrolled into PrEP* |
| --- | --- | --- | --- | --- |
|  |  |  |  |  |

- Are you familiar with immediate treatment – that is, treatment immediately after HIV diagnosis, regardless of CD4 count? What is your opinion regarding the importance of early treatment for HIV?

what are the benefits?

Any drawbacks?

- - How is it done in your health facility?
  - What would you say has been the impact if immediate treatment since it was launched in the country?
- What is your opinion regarding same day initiation of ART treatment?
  - How would you compare in terms of retention? patients initiated on treatment same day upon diagnosis and those that initiate some days later?
  - After how many days would you say patients diagnosed with HIV are put on treatment here in your facility?
- In your experience are there some patients who do decline immediate treatment?

(*What are the reasons they give? What are some of the characteristics of such patients*? *Any tension between ART and alternative medicine*?

- Any specific fears and/or concerns that patients newly diagnosed with HIV do have on immediate treatment?
- What would you say has been your greatest challenge in the implementation of immediate treatment for newly diagnosed HIV patients?

1. **PrEP provision**

*In the TMP study, partners who tested HIV negative in serodiscordant relationships and consented were put on Pre-exposure prophylaxis (PrEP). PrEP is a course of HIV drugs taken by HIV-negative people at substantial ongoing risk of HIV infection to reduce their risk of HIV acquisition.*

*(provide figures here of number of partners put on PrEP)*

**PrEP Uptake among HIV Negative Partners**

| *# of partners reported* | *# of partners tested* | *# of partners testing HIV positive* | *# of partners testing HIV negative* | *# enrolled into PrEP* |
| --- | --- | --- | --- | --- |
|  |  |  |  |  |

- What is your opinion on PrEP as a HIV prevention strategy?
- Looking at the data regarding the number of partners tested and those that were enrolled for PrEP, what would be the explanation on the numbers?

What would you say are the concerns for the people that require PrEP?

(*Probe for issues such as cost, availability, stigma, adherence, belief in effectiveness/fear of side effects/testing positive*)

- Does your health facility offer PrEP?
  - *What factors were considered for the facility to begin offering PrEP? Who is eligible for PrEP at the facility?*
  - *What has been your experience in offering PrEP? Uptake?*
- What are some of the challenges you have had to deal with while offering PrEP as a health facility?
  - *Possible barriers that health providers may encounter when offering PrEP*
  - *Challenges that health facilities may face with identifying high-risk patients and offering them PrEP*
- What support would you say health providers require in order to improve PrEP uptake?

**Other comments and recommendations**

- Any other comments you have regarding the discussion and the *Tambua Mapema Plus* study? What about any recommendations that we may have not discussed?

*Kindly thank the participants for sharing their views.*

**END**
